# Exploring the causal relationship between gut microbiota and atopic dermatitis: A Mendelian randomization study

**DOI:** 10.1097/MD.0000000000040193

**Published:** 2024-12-27

**Authors:** Wen Li, Aimin Li

**Affiliations:** a Department of Pediatrics, Jingzhou Hospital Affiliated to Yangtze University, Jingzhou, China.

**Keywords:** atopic dermatitis, causality, gut microbiota, Mendelian randomization analysis

## Abstract

Accumulating evidence indicates a correlation between gut microbiota (GM) and atopic dermatitis (AD). Nevertheless, the causal relationship between specific pathogenic bacterial taxa and AD remains uncertain. This investigation utilized a two-sample Mendelian randomization (MR) analysis to assess the causal association between GM and AD, identifying the most influential GM taxa. An MR study was conducted utilizing summary statistics derived from genome-wide association studies encompassing 207 GM taxa and their association with AD risk. The genome-wide association studies summary statistics for 207 GM taxa (from phylum to species level) were generated by the Dutch Microbiome Project. The genetic variants (*P* < 1 × 10^-5^) correlated with GM (n = 7738) were identified from this investigation. Additionally, 4 supplementary MR approaches, simple mode, MR-Egger, weighted mode, and weighted median, supported the inverse-variance weighted approach. Furthermore, sensitivity analyses were executed using leave-one-out analysis, Cochran Q test, MR-Egger intercept test, MR pleiotropy residual sum and outlier global test, and MR Steiger test. The MR analysis identified 17 distinct bacterial taxa involving 2 orders, 4 families, 5 genera, and 6 species. The inverse-variance weighted method demonstrated that 6 bacterial taxa were positively associated with AD. These taxa included the order *Pasteurellales*, family *Burkholderiales noname*, family *Pasteurellaceae*, genus *Burkholderiales noname*, species *Burkholderiales bacterium_1_1_47*, and species *Desulfovibrio piger*. Eleven bacterial taxa were negatively associated with AD, comprising the order *Actinomycetales,* family *Micrococcaceae*, family *Oscillospiraceae*, genus *Rothia*, genus *Collinsella*, genus *Oscillibacter*, genus *Pseudoflavonifractor*, species *Oscillibacter_unclassified*, species *Roseburia hominis*, species *R mucilaginosa*, and species *Parabacteroides merdae*. Moreover, the MR-Egger intercept test and MR pleiotropy residual sum and outlier global test validated that the MR analysis remained unaffected by horizontal pleiotropy (*P* > .05). Furthermore, the leave-one-out analysis contributed to validating the robustness of the outcomes. Finally, an MR Steiger directionality test confirmed the assessment of potential causal direction (*P* < .001). This investigation identified specific intestinal flora causally associated with AD risk, offering novel insights for future investigations and innovative avenues for AD diagnosis, therapeutic intervention, and prognostic assessment.

## 1. Introduction

Atopic dermatitis (AD), also referred to as eczema or atopic eczema, manifests as a prevalent allergic condition marked by chronic skin inflammation. Globally, AD impacts approximately 15% to 20% of children and 5% to 10% of adults, with its prevalence steadily increasing worldwide.^[1–3]^ The disease presents with recurring eczema lesions and severe itching, exerting a substantial psychosocial impact on patients and relatives.^[4]^ AD emerges during infancy, commonly between 3 to 6 months of age, with approximately 60% of children experiencing onset by their first year and around 90% experiencing it by 5 years of age.^[5–7]^ In some severely affected individuals, the disease persists into adulthood, while a minority of them experience its first onset during adulthood.^[8]^ Early childhood AD frequently serves as an early indicator of subsequent asthma and/or allergic rhinitis.^[9]^ The clinical diagnosis of AD lacks definitive laboratory tests. However, utilizing previously validated diagnostic criteria, The American Academy of Dermatology has refined the diagnostic process to improve its applicability in diagnosing AD across different age groups, including infants, children, and adults.^[10,11]^ For further details, refer to Table S1, Supplemental Digital Content, http://links.lww.com/MD/N774.

The pathogenesis of AD is complex and multifaceted, encompassing immunological, genetic, and environmental factors. It results in skin barrier impairment and immune dysregulation. Primary pathogenic mechanisms entail epidermal barrier dysfunction and type 2 dominant skin inflammation.^[12]^ The “gut–skin axis^[13]^” has garnered increasing attention, with numerous investigations proposing that alterations in the composition and diversity of the gut microbiota (GM) can impact skin immunity and metabolism.^[14,15]^ The GM encompasses microorganisms within the gut of the host that not only regulate metabolism but also exert a significant influence on both local and systemic immunity.^[16]^ GM establishes a dynamic ecological equilibrium between the external environment and the host. Moreover, the microbiota either directly or indirectly regulates all immune system components. Animal experiments have demonstrated that GM influences local and systemic immunity.^[17]^ Disruption in either the microbiota or the host response can initiate chronic inflammation. Reduced diversity of GM is correlated with dysbiosis of gut ecology, which can cause various diseases, including allergies.^[18]^ Polkowska-Pruszyska et al^[19]^ elucidated the correlation between alterations in the GM community and the onset of immune-mediated diseases, such as acne vulgaris, rosacea, psoriasis, psoriatic arthritis, AD, and others.

However, the precise roles of several GM taxa in AD require further investigation. Like randomized controlled trials, Mendelian randomization (MR) is a data analysis strategy utilized to evaluate etiological inferences in epidemiological research. It utilizes genetic variants strongly correlated to the exposure factors as instrumental variables (IVs) to assess the causal relationship between exposure factors and outcomes.^[20,21]^ Genome-wide association studies (GWAS) represent an innovative approach that employs millions of single nucleotide polymorphisms (SNPs) in the genome as molecular genetic markers. This strategy enables genome-wide controlled or correlation analyses to identify genetic variants influencing complex traits through comparative analyses.^[22]^ SNPs are primarily deoxyribonucleic acid sequence polymorphisms due to alterations in a single nucleotide at the genomic level. SNPs adhere to the principle of random allocation of genetic variants during meiosis. This procedure mitigates the involvement of confounding factors and diminishes the likelihood of reverse causation, as genetic variants precede the onset of diseases.^[23]^

This investigation employed MR analysis utilizing large-scale GWAS summary statistics of GM and AD to evaluate potentially influential GM taxa. The outcomes could help validate existing data on AD and offer novel insights for its prevention and treatment.

## 2. Materials and methods

### 2.1. Study design

**Figure 1.**
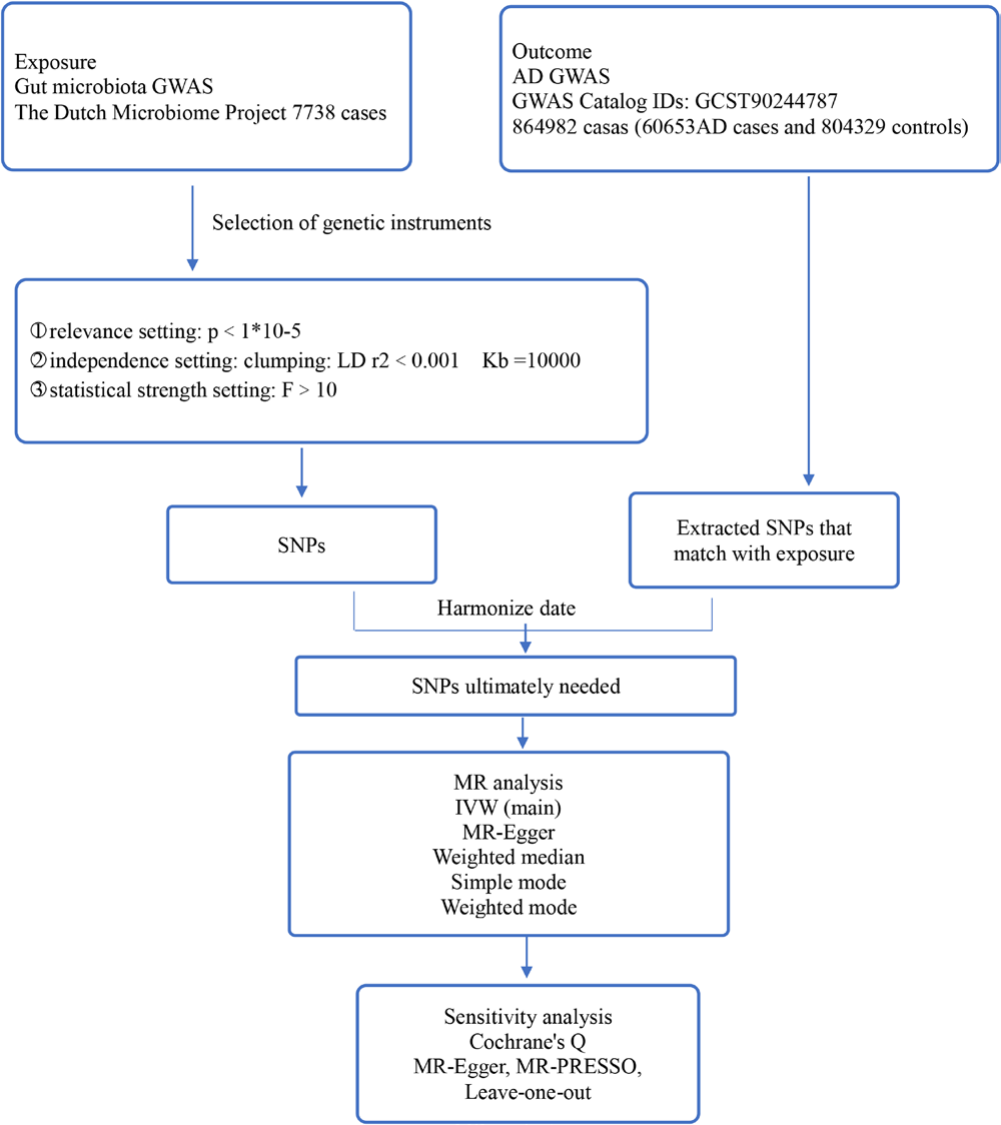
Flowchart representing the overview of the study. AD = atopic dermatitis; GWAS = genome-wide association study; IVW = Inverse variance weighted; LD = linkage disequilibrium; MR: Mendelian randomization; SNPs = single nucleotide polymorphisms.

The overview of the study design is depicted in Figure 1. MR analysis must satisfy the 3 assumptions: 1. Genetic instrumental variants must demonstrate a strong link with exposure. 2. Genetic variants utilized should not be associated with any potential confounding factors. 3. The selected genetic variants should impact outcomes solely through the exposure pathway.^[23,24]^ This investigation determined GM taxa that have a causal impact on AD by conducting a two-sample MR analysis.

### 2.2. Data sources for the exposure

The GM summary statistics were extracted from GWAS data encompassing 207 bacterial taxa (from phylum to species level) and 205 pathways reflecting microbial composition and function in 7738 samples of the Dutch Microbiome Project.^[25]^ These summary statistics of GMs are available at (Dutch Microbiome Project [molgeniscloud.org]). Furthermore, the Integrative Epidemiology Unit ID numbers (Integrative Epidemiology Unit OpenGWAS project [mrcieu.ac.uk]) corresponding to the abundances of the 207 GM taxa were sourced from datasets labeled ebi-a-GCST90027651 to ebi-a-GCST90027857.

### 2.3. Data sources for the outcome

The GWAS data for AD was procured from the latest version of summary statistics data released in the GWAS Catalog in October 2023. Specifically, the data retrieved corresponds to the study accession ID GCST90244787 (GWAS Catalog [ebi.ac.uk]). This investigation represents the largest GWAS on AD, incorporating a meta-analysis of European populations (N = 864,982; 60,653 AD cases and 804,329 controls from 40 cohorts). It was successful in determining 81 genome-wide substantially independently associated loci, of which 29 are novel. Comprehensive details can be found in the original article.^[26]^

### 2.4. Instrumental variables selection

This MR study employed SNPs strongly correlated with GM taxa as IVs. Given the limited number of IVs derived using a stringent threshold (*P* < 5 × 10^‐8^), a more lenient threshold (*P* < 1 × 10^‐5^) was adopted to procure a comparatively larger set of IVs.^[27–29]^ Furthermore, to verify the independence of each IV, the linkage disequilibrium parameter, *r*^2^, of SNPs was established at 0.001, with the genetic distance set at 10,000 kb. In addition, palindromic SNPs were excluded from the study. A final requirement for the study necessitated that the *F*-statistic exceeds a minimum threshold of 10 to mitigate bias arising from weak IVs. The *F*-statistic was calculated utilizing the formula: *F* = *R*^2^(N-*k*-1)/*k*(1‐*R*^2^), where *R*^2^ denotes the coefficient of determination, N denotes the sample size, and *k* signifies the number of included SNPs. IVs with *F*-values below 10 were omitted to ensure the strength of the correlation between IVs and exposure factors.^[30]^

### 2.5. Statistical analysis

The primary analysis in this investigation employed the inverse-variance weighted (IVW) model, which is based on the meta-analysis principle combined with the estimation of the Wald ratio for each SNP. Moreover, 4 other MR strategies, encompassing weighted median, MR-Egger regression, simple mode, and weighted mode, were utilized to complement the IVW outcomes.^[20,31,32]^

Sensitivity analyses were executed to analyze the strength of the causal relationship. This included 2 methods for detecting and correcting horizontal pleiotropy: the MR-Egger intercept test^[33]^ and the MR pleiotropy residual sum and outlier global test.^[34]^ Pleiotropy was assessed by utilizing the *P*-value of the pleiotropy test. A *P*-value > .05 indicated weak pleiotropy in the causal effect, which was disregarded.^[32]^ Moreover, the outcomes of causal relationships were depicted as odds ratios (OR) and 95% confidence intervals (95% CI), with the significance threshold set at *P* < .05. Additionally, Cochran IVW Q test was executed to assess the heterogeneity of IVs.^[35]^ Finally, a leave-one-out sensitivity analysis was executed to evaluate the influence of individual SNPs on the overall estimates. Additionally, the MR Steiger test was employed to quantify the variance attributable to SNPs on both outcome and exposure. If the variance accounted for by the outcome is less than that of exposure, then the direction of the effect is considered to be correct. This examination assists in assessing the suitability of the direction of the analysis. A small *P*-value indicates the absence of reverse causality and thus provides evidence of the stability and reliability of the outcomes.^[36]^

The analyses in this investigation were executed utilizing R software (v 4.3.1). “TwoSampleMR” and “MRPRESSO” were utilized in this MR analysis.

### 2.6. Ethical approval, data availability statement

All data used in the current study are publicly available GWAS summary data. All original GWAS studies were subject to ethical review and approval. Consequently, no further ethical approval was required.

## 3. Results

### 3.1. Instrumental variable data

The GM data comprised 207 bacterial taxa traits, categorized into 6 biological classifications: phylum, class, order, family, genus, and species. After accounting for strong correlations and excluding SNPs with linkage disequilibrium effects, the IVs were subjected to MR analysis. This process resulted in the identification of 127 independent SNPs within specific bacterial taxa linked to AD. The relevant information on SNPs, comprising beta (β), standard error, OR, and *P*-value, was systematically compiled for subsequent investigation. Furthermore, the *F*-statistic for all SNPs surpassed 10, implying that the estimates were unlikely to exhibit weak instrumental bias. The selected IVs are detailed in Table S2, Supplemental Digital Content, http://links.lww.com/MD/N774.

### 3.2. Mendelian randomization analysis

**Figure 2.**
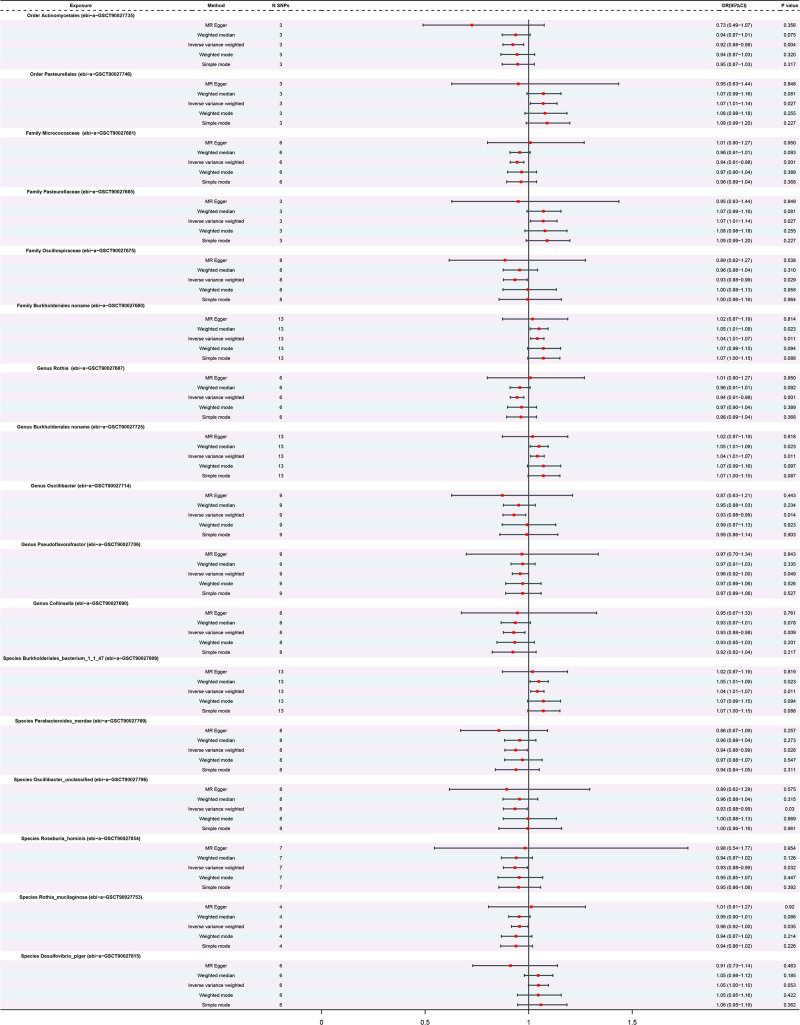
Forest plot for causal association between bacterial taxa and AD. AD = atopic dermatitis, CI = confidence interval; OR = odds ratio; *P* < .05 is the nominal significance.

In this investigation, 17 bacterial taxa were linked to AD (Fig. 2 and Table S3, Supplemental Digital Content, http://links.lww.com/MD/N774). Six of these taxa exhibited a positive correlation with AD, as confirmed by IVW analysis. This included the order *Pasteurellales* (ID: ebi-a-GCST90027746, OR = 1.07, *P* = .026718), family *Burkholderiales noname* (ID: ebi-a-GCST90027680, OR = 1.04, *P* = .01055), family *Pasteurellaceae* (ID: ebi-a-GCST90027685, OR = 1.07, *P* = .026705), genus *Burkholderiales noname* (ID: ebi-aGCST90027725, OR = 1.04, *P* = .01058), species *Burkholderiales_bacterium_1_1_47* (ID: ebi-a-GCST90027809, OR = 1.04, *P* = .01059), and species *Desulfovibrio piger* (ID: ebi-a-GCST90027815, OR = 1.05, *P* = .052547).

Conversely, 11 bacterial taxa exhibited a negative correlation with AD, which included the order *Actinomycetales* (ID: ebi-a-GCST90027735, OR = 0.92, *P* = .004374), family *Micrococcaceae* (ID: ebi-a-GCST90027661, OR = 0.94, *P* = .001222), family *Oscillospiraceae* (ID: ebi-a-GCST90027675, OR = 0.93, *P* = .028954), genus *Rothia* (ID: ebi-a-GCST90027687, OR = 0.94, *P* = .001219), genus *Collinsella* (ID: ebi-a-GCST90027690, OR = 0.93, *P* = .00885), genus *Oscillibacter* (ID: ebi-a-GCST90027714, OR = 0.93, *P* = .014388), genus *Pseudoflavonifractor* (ID: ebi-a-GCST90027706, OR = 0.96, *P* = .049269), species *Oscillibacter_unclassified* (ID: ebi-a-GCST90027796, OR = 0.93, *P* = .030058), species *Roseburia_hominis* (ID: ebi-a-GCST90027854, OR = 0.93, *P* = .031859), species *Rothia_mucilaginosa* (ID: ebi-a-GCST90027753, OR = 0.96, *P* = .034946), and species *Parabacteroides_merdae* (ID: ebi-a-GCST90027769, OR = 0.94, *P* = .027584).

Additionally, weighted median estimator analysis revealed that the associations for the family Burkholderiales noname (*P* = .022591), genus *Burkholderiales noname* (*P* = .022713), species *Burkholderiales bacterium_1_1_47* (*P* = .022989) were consistent with those observed utilizing IVW (*P* < .05) (Fig. 2 and Figs. S5–S7, Supplemental Digital Content, http://links.lww.com/MD/N774).

### 3.3. Sensitivity analyses

As illustrated in Table S3, Supplemental Digital Content, http://links.lww.com/MD/N774, the outcomes of sensitivity analysis from Cochran Q test demonstrated no significant heterogeneity (*P* > .05). The MR-Egger intercept and MR pleiotropy residual sum and outlier tests confirmed the absence of pleiotropy or outliers (*P* > .05). Additionally, the leave-one-out analysis revealed no SNPs with a notable impact on causality, demonstrating the strength of the MR findings (Figs. S1–S17, Supplemental Digital Content, http://links.lww.com/MD/N774). Ultimately, the MR Steiger test affirmed that all 17 specific bacterial taxa exhibited low *P*-values (*P* < .001), validating the potential causality (Table S4, Supplemental Digital Content, http://links.lww.com/MD/N774).

## 4. Discussion

The current investigation utilized the GM summary statistics retrieved from the most extensive GWAS meta-analysis of the Dutch Microbiome Project. This investigation aimed to assess the association between gut flora and AD across 6 taxonomic levels: phylum, class, order, family, genus, and species. The IVW outcomes revealed that 6 bacterial taxa, among them the family *Pasteurellaceae* (ID: ebi-a-GCST90027685, OR = 1.07, 95% CI: 1.01–1.14, *P* = .026705) and species *Burkholderiales bacterium_1_1_47* (ID: ebi-a-GCST90027809, OR = 1.04, 95% CI: 1.01–1.07, *P* = .01059), were linked to a heightened risk of AD. Simultaneously, 11 bacterial taxa were linked to a reduced risk for AD, among which the genus *Rothia* (ID: ebi-a-GCST90027687, OR = 0.94, 95% CI: 0.91–0.98, *P* = .001219) displayed a pronounced causative association with a decreased risk of AD.

AD manifests as a chronic, relapsing, and remitting inflammatory skin condition attributed to a multifaceted interaction involving immune dysregulation, epidermal gene mutations, and environmental influences. These factors result in epidermal damage and intensely pruritic skin lesions.^[37]^ Advancing research on the gut–skin axis has elucidated that dysbiosis of GM can lead to immune and metabolic disorders of the skin, thereby promoting inflammatory skin diseases.^[13]^ Skin lesions observed in AD typically exhibit reduced bacterial diversity.^[38]^ Numerous recent investigations have revealed associations between AD and alterations in gut flora composition.^[19]^

Metabolites of the intestinal flora, encompassing acetate, propionate, and butyrate, known as short-chain fatty acids, contribute to immune system regulation and the modulation of inflammatory responses. They achieve this by impeding inflammatory cell proliferation and cytokine production, thus suppressing immune reactions.^[39]^ Short-chain fatty acids can enhance the energy levels of the intestinal epithelium and improve the integrity of the intestinal epithelial barrier. These effects are achieved by preventing the entry of microbes and toxins into the humoral circulation, thereby triggering T-helper type 2 immunity. Such immune responses, if activated, are known to disrupt skin homeostasis.^[40]^

GM is predominantly composed of *Bacteroidetes*, *Firmicutes*, *Actinobacteria*, and *Proteobacteria* at the phylum level, which exhibit relative stability. Nevertheless, there is substantial variation in the bacterial composition at the species level.^[41]^

The phylum *Proteobacteria* is usually classified into 5 classes based on their ribosomal ribonucleic acid sequences, designated by the Greek letters α, β, γ, δ, and ε.^[42]^ Shin et al^[43]^ noted that an elevation in the abundance of the phylum *Proteobacteria* is a marker for an unstable microbial community (dysbiosis) and may serve as a potential diagnostic criterion for disease. In the present investigation, the phylum *Proteobacteria*, encompassing the classes *Betaproteobacteria*, *Gammaproteobacteria*, and *Deltaproteobacteria*, was linked to an elevated risk of AD. The genus *Burkholderia* is classified under *Betaproteobacteria*, whereas the order *Pasteurellales*, which includes the family *Pasteurellaceae*, belongs to *Gammaproteobacteria.* The genus *Desulfovibrio* falls under the class *Deltaproteobacteria*. All these bacteria are intestinal pathogens. They have the potential to induce intestinal dysfunction, resulting in dysbiosis, which in turn indirectly heightens the risk of AD.

*Firmicutes* represent a major phylum within the normal human GM.^[44]^ One of its species, *Roseburia hominis*, is a specialized Gram-positive anaerobic bacterium categorized under the genus *Roseburia.* It can produce butyrate through substrate-level phosphorylation and proton gradients, which are crucial for intestinal epithelial energy generation and the modulation of cellular responses.^[45]^ The findings of this investigation indicate a causal relationship between the decreased relative abundance of *R hominis* and an elevated risk of AD.

*Clostridiales* is categorized under the phylum *Firmicutes* and encompasses the order *Clostridia,* which harbors a diverse array of families, genera, and species. Most *Clostridium* species are nonpathogenic, with the genus *Clostridium butyricum*, also known as MIYAIRI (MIYAIRI 588), as a notable representative. This particular strain functions as an intestinal probiotic with potent rectifying effects, suppressing pathogenic bacteria within the intestinal tract while promoting the growth of beneficial bacteria such as *Bifidobacterium* and *Lactobacillu*s.^[46]^ Wang et al revealed that in contrast to the normal group, the relative abundance of genus *Clostridium_sensu_stricto_1* was decreased in individuals with AD. This genus comprises various species, among them is *C butyricum*, renowned for its capacity to synthesize butyric acid, an SCFA recognized for conferring benefits to gut health.^[47]^ The outcomes of this investigation suggest that the intestinal flora of the family *Oscillospiraceae*, genus *Oscillibacter* and its subordinate species *Oscillibacter unclassified*, and family *Clostridiaceae* and its subordinate genus *Pseudoflavonifractor* exhibited a negative correlation with AD occurrence. These outcomes indicate that this group of bacteria may be a protective factor against AD. Moreover, Xue et al revealed that *Tenericutes*, *Mollicutes*, *Clostridia*, and *Bifidobacteriales* were negatively correlated with the risk of AD.^[48]^ Similarly, Zhao et al demonstrated that azithromycin pretreatment depleted SCAF-producing bacterial genera (*Alistipes*, *Clostridiales_unclassified*, *Butyricoccus*) and exacerbated trimellitic anhydride-induced AD-like symptoms in mice.^[49]^ Lee et al reported a higher alpha diversity of the gut microbiome in infant atopic eczema.^[50]^ Ewa Łoś-Rycharska et al noted that approximately 15% to 30% of children with AD also suffer from food allergies,^[51]^ a condition associated with lower abundance and diversity of lactic acid bacteria and *Clostridia*.^[52]^ Martin et al investigated gut longitudinal disease-associated microbiome variations in children with food protein-induced allergic proctocolitis (FPIAP). The outcomes of the research revealed that infants with FPIAP had a decreased abundance of the *Clostridiales* order during the symptomatic period. As commonly known, FPIAP is the earliest recognized non-immunoglobulin E (IgE)-mediated food allergy in infancy, often associated with AD.^[53]^ These findings align with the outcomes of the present investigation.

*Actinomycetes* and *Bacteroidetes* are also key components of the intestinal flora and are vital in regulating intestinal homeostasis. West et al reported that the lowered relative abundance of potentially regulatory gut bacteria is linked to excessive inflammatory cytokine responses to toll-like receptor (TLR) ligands (TLR2 and TLR4) and the occurrence of IgE-associated eczema. The outcomes indicated that mothers of infants with IgE-associated eczema had reduced α-diversity in *Bacteroidetes*. While at the age of 1 year, children with IgE-associated eczema had decreased α-diversity of Ac*tinobacteria*, in comparison to the control group.^[54]^

*Coriobacteriaceae* is a family of bacteria in the order *Coriobacteriales*, which contains the genera *Coriobacterium* and *Collinsella*, both of which belong to the phylum *Actinobacteria*. Additionally, *Parabacteroides,* a genus of Gram-negative, anaerobic bacteria, falls under the phylum *Bacteroidetes*. It represents a prevalent component of the human intestinal microflora, including the species *Parabacteroides merdae*. Two recent investigations in children and adults with AD noted that *Parabacteroides* and *Bacteroides* were abundant in the group with severe AD.^[55,56]^ Similarly, Liu et al explored the association between alterations in the gut microbiome and the severity of AD in infants. The outcomes revealed elevated levels of *Parabacteroides* and reduced levels of *Collinsella* in both the moderate and severe AD groups.^[57]^ Moreover, Wang et al assessed the impact of a novel E3 probiotic formulation on the GM of individuals with AD. A decrease in the relative abundance of *Collinsella*, *Fusicatenibacter*, *Erysipelatoclostridium*, *Bilophila*, and other bacteria was observed following oral administration of the probiotic mixture. This reveals that the relatively elevated abundance of these bacteria in individuals with AD may exacerbate AD symptoms.^[58]^ However, in the present investigation, the genus *Collinsella* and species *Parabacteroides merdae* were negatively associated with the risk of AD, suggesting a potential protective role in developing AD. These outcomes are inconsistent with other studies and warrant further investigation in future research.

*Rothia* is a genus of Gram-positive bacteria that includes the species *Rothia mucilaginosa*. *Rothia* is part of the family *Micrococcaceae* within the order *Actinomycetales.* These bacteria typically inhabit the human mouth and throat and can be conditionally pathogenic. In an investigation by Hong et al, it was noted that the diversity of gut flora was reduced in children with autism comorbid with AD. However, in controls without AD comorbidity, the abundance of *R mucilaginosa* was higher.^[59]^ This outcome aligns with the findings of the present investigation, indicating that an increased relative abundance of *R mucilaginosa* is causally correlated with a lower risk of AD.

This investigation establishes a causal relationship between specific bacterial taxa and AD, providing new insights into the management of AD. Probiotics have been proposed as therapeutic and preventive interventions for allergic diseases.^[60]^ Among the most commonly utilized species are *Lactobacillus* and *Bifidobacterium,* which are involved in reducing the risk of AD.^[61]^ However, the precise efficacy or preventive role of probiotics is still controversial and necessitates further investigation.^[62–64]^

The present study exhibits specific strengths. The chosen genetic variables were sourced from the recent Dutch Microbiome Project GWAS data summary of 412 GM groups (comprising 207 GM taxa and 205 Gut bacterial pathway). It enables a comprehensive analysis of the impact of bacterial taxa on outcomes ranging from the level of phylum to species. This represents a pioneering effort compared to prior studies. Secondly, the data for AD were derived from summary statistics of a large-scale GWAS, encompassing 60,653 patients and 804,329 controls from 40 cohorts. This substantial sample size allowed for a wide range of sensitive analyses to be conducted, ensuring the robustness and accuracy of the results. Finally, MR analyses, adhering to the principle of random allocation of parental alleles to offspring, mimic randomized controlled trials and are not susceptible to conventional confounders. This statistical advantage aids in minimizing the influences of bias and reverse causation.

However, the investigation also presents certain limitations. Firstly, because GWAS primarily relies on data from individuals of European ancestry for exposure and outcome statistics, it may not fully reflect the genetic diversity present in other racial and ethnic groups. Due to the range of environmental, dietary, and other factors, different races do not have the same predominant intestinal flora, potentially introducing bias in the results. Secondly, AD is a chronic inflammatory skin disease with a complex etiology. The cases analyzed lacked specific sample data, resulting in the inability to perform subgroup analyses based on age and gender. Finally, the outcomes of the present investigation require further validation through clinical and basic research. The link between GM and inflammatory skin diseases should be explored in diverse populations and at a more detailed species level.

## 5. Conclusion

This research used a two-sample MR analysis to evaluate the causal correlation between GM and AD. The analysis employed GM summary statistics from the most extensive GWAS meta-analysis executed by the Dutch Microbiome Project and AD summary statistics from the GWAS Catalog. Seventeen bacterial taxa were identified as causally associated with AD. Among them, 6 bacterial taxa exhibited a positive association with AD, while 11 bacterial taxa exhibited a negative association with AD. Notably, the genus *Rothia* (ID: ebi-a-GCST90027687, OR = 0.94, 95% CI: 0.91–0.98, *P* = .001219) displayed a strong causal relationship with a reduced risk of AD. This investigation determined specific microbiota employing genetic prediction, which could serve as valuable biomarkers for early disease diagnosis and as promising therapeutic measures for AD.

## Acknowledgments

We wish to thank the investigators for sharing the GWAS data used in the present study and the Dutch Microbiome Project for providing the GWAS data on intestinal flora. We also thank Bullet Edits Limited for the linguistic editing and proofreading of the manuscript.

## Author contributions

**Writing – original draft:** Wen Li.

**Writing – review & editing:** Aimin Li.

## Supplementary Material



## References

[1] Asher MI Montefort S Björkstén B. Worldwide time trends in the prevalence of symptoms of asthma, allergic rhinoconjunctivitis, and eczema in childhood: ISAAC phases one and three repeat multicountry cross-sectional surveys. Lancet. 2006;368:733–43. 16935684 10.1016/S0140-6736(06)69283-0

[2] Langan SM Irvine AD Weidinger S. Atopic dermatitis. Lancet. 2020;396:345–60. 32738956 10.1016/S0140-6736(20)31286-1

[3] Strom MA Silverberg JI. Utilization of preventive health care in adults and children with eczema. Am J Prev Med. 2016;50:e33–44. 26547540 10.1016/j.amepre.2015.07.029 PMC5237391

[4] Frazier W Bhardwaj N. Atopic dermatitis: diagnosis and treatment. Am Fam Physician. 2020;101:590–8. 32412211

[5] Abuabara K Hoffstad O Troxel AB Gelfand JM McCulloch CE Margolis DJ. Patterns and predictors of atopic dermatitis disease control past childhood: an observational cohort study. J Allergy Clin Immunol. 2018;141:778–80.e6. 28629748 10.1016/j.jaci.2017.05.031 PMC6532763

[6] Barnetson RSC Rogers M. Childhood atopic eczema. BMJ. 2002;324:1376–9. 12052810 10.1136/bmj.324.7350.1376 PMC1123328

[7] Kay J Gawkrodger DJ Mortimer MJ Jaron AG. The prevalence of childhood atopic eczema in a general population. J Am Acad Dermatol. 1994;30:35–9. 8277028 10.1016/s0190-9622(94)70004-4

[8] Ellis CN Mancini AJ Paller AS Simpson EL Eichenfield LF. Understanding and managing atopic dermatitis in adult patients. Semin Cutan Med Surg. 2012;31(3 Suppl):S18–22. 23021781 10.1016/j.sder.2012.07.006

[9] Spergel JM. From atopic dermatitis to asthma: the atopic march. Ann Allergy Asthma Immunol. 2010;105:99–106; quiz 107. 20674819 10.1016/j.anai.2009.10.002

[10] Eichenfield LF Hanifin JM Luger TA Stevens SR Pride HB. Consensus conference on pediatric atopic dermatitis. J Am Acad Dermatol. 2003;49:1088–95. 14639390 10.1016/s0190-9622(03)02539-8

[11] Eichenfield LF Tom WL Chamlin SL. Guidelines of care for the management of atopic dermatitis: section 1. Diagnosis and assessment of atopic dermatitis. J Am Acad Dermatol. 2014;70:338–51. 24290431 10.1016/j.jaad.2013.10.010 PMC4410183

[12] Boguniewicz M Leung DYM. Atopic dermatitis: a disease of altered skin barrier and immune dysregulation. Immunol Rev. 2011;242:233–46. 21682749 10.1111/j.1600-065X.2011.01027.x PMC3122139

[13] De Pessemier B Grine L Debaere M Maes A Paetzold B Callewaert C. Gut–skin axis: current knowledge of the interrelationship between microbial dysbiosis and skin conditions. Microorganisms. 2021;9:353. 33670115 10.3390/microorganisms9020353 PMC7916842

[14] Mahmud MR Akter S Tamanna SK. Impact of gut microbiome on skin health: gut–skin axis observed through the lenses of therapeutics and skin diseases. Gut Microbes. 2022;14:2096995. 35866234 10.1080/19490976.2022.2096995 PMC9311318

[15] Wang M Karlsson C Olsson C. Reduced diversity in the early fecal microbiota of infants with atopic eczema. J Allergy Clin Immunol. 2008;121:129–34. 18028995 10.1016/j.jaci.2007.09.011

[16] Thaiss CA Zmora N Levy M Elinav E. The microbiome and innate immunity. Nature. 2016;535:65–74. 27383981 10.1038/nature18847

[17] Wang H Wang G Banerjee N. Aberrant gut microbiome contributes to intestinal oxidative stress, barrier dysfunction, inflammation and systemic autoimmune responses in MRL/lpr Mice. Front Immunol. 2021;12:651191. 33912174 10.3389/fimmu.2021.651191 PMC8071869

[18] Zimmermann P Messina N Mohn WW Finlay BB Curtis N. Association between the intestinal microbiota and allergic sensitization, eczema, and asthma: a systematic review. J Allergy Clin Immunol. 2019;143:467–85. 30600099 10.1016/j.jaci.2018.09.025

[19] Polkowska-Pruszyńska B Gerkowicz A Krasowska D. The gut microbiome alterations in allergic and inflammatory skin diseases—an update. J Eur Acad Dermatol Venereol. 2020;34:455–64. 31520544 10.1111/jdv.15951

[20] Pagoni P Dimou NL Murphy N Stergiakouli E. Using Mendelian randomisation to assess causality in observational studies. Evid Based Ment Health. 2019;22:67–71. 30979719 10.1136/ebmental-2019-300085 PMC10270458

[21] Swanson SA Tiemeier H Ikram MA Hernán MA. Nature as a Trialist?: Deconstructing the analogy between Mendelian randomization and randomized trials. Epidemiology. 2017;28:653–9. 28590373 10.1097/EDE.0000000000000699 PMC5552969

[22] Visscher PM Brown MA McCarthy MI Yang J. Five years of GWAS discovery. Am J Hum Genet. 2012;90:7–24. 22243964 10.1016/j.ajhg.2011.11.029 PMC3257326

[23] Lawlor DA Harbord RM Sterne JAC Timpson N Davey Smith G. Mendelian randomization: using genes as instruments for making causal inferences in epidemiology. Stat Med. 2008;27:1133–63. 17886233 10.1002/sim.3034

[24] Burgess S Small DS Thompson SG. A review of instrumental variable estimators for Mendelian randomization. Stat Methods Med Res. 2017;26:2333–55. 26282889 10.1177/0962280215597579 PMC5642006

[25] Lopera-Maya EA Kurilshikov A van der Graaf A. Effect of host genetics on the gut microbiome in 7,738 participants of the Dutch Microbiome Project. Nat Genet. 2022;54:143–51. 35115690 10.1038/s41588-021-00992-y

[26] Budu-Aggrey A Kilanowski A Sobczyk MK. European and multi-ancestry genome-wide association meta-analysis of atopic dermatitis highlights importance of systemic immune regulation. Nat Commun. 2023;14:6172. 37794016 10.1038/s41467-023-41180-2 PMC10550990

[27] You J Bi X Zhang K. Causal associations between gut microbiota and sepsis: a two-sample Mendelian randomization study. Eur J Clin Invest. 2023;53:e14064. 37464539 10.1111/eci.14064

[28] Zeng Y Cao S Yang H. Roles of gut microbiome in epilepsy risk: a Mendelian randomization study. Front Microbiol. 2023;14:1115014. 36922970 10.3389/fmicb.2023.1115014 PMC10010438

[29] Zhong Y Wang F Meng X Zhou L. The associations between gut microbiota and inflammatory skin diseases: a bi-directional two-sample Mendelian randomization study. Front Immunol. 2024;15:1297240. 38370414 10.3389/fimmu.2024.1297240 PMC10869565

[30] Pierce BL Ahsan H Vanderweele TJ. Power and instrument strength requirements for Mendelian randomization studies using multiple genetic variants. Int J Epidemiol. 2011;40:740–52. 20813862 10.1093/ije/dyq151 PMC3147064

[31] Bowden J Davey Smith G Haycock PC Burgess S. Consistent estimation in mendelian randomization with some invalid instruments using a weighted median estimator. Genet Epidemiol. 2016;40:304–14. 27061298 10.1002/gepi.21965 PMC4849733

[32] Burgess S Thompson SG. Interpreting findings from Mendelian randomization using the MR-Egger method. Eur J Epidemiol. 2017;32:377–89. 28527048 10.1007/s10654-017-0255-x PMC5506233

[33] Rees JMB Wood AM Burgess S. Extending the MR-Egger method for multivariable Mendelian randomization to correct for both measured and unmeasured pleiotropy. Stat Med. 2017;36:4705–18. 28960498 10.1002/sim.7492 PMC5725762

[34] Verbanck M Chen C-Y Neale B Do R. Detection of widespread horizontal pleiotropy in causal relationships inferred from Mendelian randomization between complex traits and diseases. Nat Genet. 2018;50:693–8. 29686387 10.1038/s41588-018-0099-7 PMC6083837

[35] Greco MFD Minelli C Sheehan NA Thompson JR. Detecting pleiotropy in Mendelian randomisation studies with summary data and a continuous outcome. Stat Med. 2015;34:2926–40. 25950993 10.1002/sim.6522

[36] Xiao G He Q Liu L. Causality of genetically determined metabolites on anxiety disorders: a two-sample Mendelian randomization study. J Transl Med. 2022;20:475. 36266699 10.1186/s12967-022-03691-2 PMC9583573

[37] Abuabara K Magyari A McCulloch CE Linos E Margolis DJ Langan SM. Prevalence of atopic eczema among patients seen in primary care: data from the health improvement network. Ann Intern Med. 2019;170:354–6. 30508419 10.7326/M18-2246 PMC6548682

[38] Bjerre RD Bandier J Skov L Engstrand L Johansen JD. The role of the skin microbiome in atopic dermatitis: a systematic review. Br J Dermatol. 2017;177:1272–8. 28207943 10.1111/bjd.15390

[39] Martin-Gallausiaux C Marinelli L Blottière HM Larraufie P Lapaque N. SCFA: mechanisms and functional importance in the gut. Proc Nutr Soc. 2021;80:37–49. 32238208 10.1017/S0029665120006916

[40] O’Neill CA Monteleone G McLaughlin JT Paus R. The gut–skin axis in health and disease: a paradigm with therapeutic implications. Bioessays. 2016;38:1167–76. 27554239 10.1002/bies.201600008

[41] Hillman ET Lu H Yao T Nakatsu CH. Microbial ecology along the gastrointestinal tract. Microbes Environments. 2017;32:300–13. 29129876 10.1264/jsme2.ME17017 PMC5745014

[42] The Human Microbiome Project Consortium. Structure, function and diversity of the healthy human microbiome. Nature. 2012;486:207–14. 22699609 10.1038/nature11234 PMC3564958

[43] Shin N-R Whon TW Bae J-W. Proteobacteria: microbial signature of dysbiosis in gut microbiota. Trends Biotechnol. 2015;33:496–503. 26210164 10.1016/j.tibtech.2015.06.011

[44] Jandhyala SM Talukdar R Subramanyam C Vuyyuru H Sasikala M Nageshwar Reddy D. Role of the normal gut microbiota. World J Gastroenterol. 2015;21:8787–803. 26269668 10.3748/wjg.v21.i29.8787 PMC4528021

[45] Machiels K Joossens M Sabino J. A decrease of the butyrate-producing species *Roseburia hominis* and *Faecalibacterium prausnitzii* defines dysbiosis in patients with ulcerative colitis. Gut. 2014;63:1275–83. 24021287 10.1136/gutjnl-2013-304833

[46] Ariyoshi T Hagihara M Eguchi S. *Clostridium butyricum* MIYAIRI 588-induced protectin D1 has an anti-inflammatory effect on antibiotic-induced intestinal disorder. Front Microbiol. 2020;11:587725. 33193245 10.3389/fmicb.2020.587725 PMC7661741

[47] Wang Y Hou J Tsui JC-C. Unique gut microbiome signatures among adult patients with moderate to severe atopic dermatitis in Southern Chinese. Int J Mol Sci . 2023;24:12856. 37629036 10.3390/ijms241612856 PMC10454836

[48] Xue Y Zhang L Chen Y Wang H Xie J. Gut microbiota and atopic dermatitis: a two-sample Mendelian randomization study. Front Med (Lausanne). 2023;10:1174331. 37425302 10.3389/fmed.2023.1174331 PMC10323683

[49] Zhao H Zhou J Lu H. Azithromycin pretreatment exacerbates atopic dermatitis in trimellitic anhydride-induced model mice accompanied by correlated changes in the gut microbiota and serum cytokines. Int Immunopharmacol. 2022;102:108388. 34819259 10.1016/j.intimp.2021.108388

[50] Lee E Lee S-Y Kang M-J. Clostridia in the gut and onset of atopic dermatitis via eosinophilic inflammation. Ann Allergy Asthma Immunol. 2016;117:91–2.e1. 27179583 10.1016/j.anai.2016.04.019

[51] Silverberg JI Simpson EL. Association between severe eczema in children and multiple comorbid conditions and increased healthcare utilization. Pediatr Allergy Immunol. 2013;24:476–86. 23773154 10.1111/pai.12095 PMC4397968

[52] Łoś-Rycharska E Gołębiewski M Grzybowski T Rogalla-Ładniak U Krogulska A. The microbiome and its impact on food allergy and atopic dermatitis in children. Postepy Dermatol Alergol. 2020;37:641–50. 33240001 10.5114/ada.2019.90120 PMC7675070

[53] Martin VM Virkud YV Dahan E. Longitudinal disease-associated gut microbiome differences in infants with food protein-induced allergic proctocolitis. Microbiome. 2022;10:154. 36138438 10.1186/s40168-022-01322-y PMC9503280

[54] West CE Rydén P Lundin D Engstrand L Tulic MK Prescott SL. Gut microbiome and innate immune response patterns in IgE-associated eczema. Clin Exp Allergy. 2015;45:1419–29. 25944283 10.1111/cea.12566

[55] Reddel S Del Chierico F Quagliariello A. Gut microbiota profile in children affected by atopic dermatitis and evaluation of intestinal persistence of a probiotic mixture. Sci Rep. 2019;9:4996. 30899033 10.1038/s41598-019-41149-6 PMC6428866

[56] Ye S Yan F Wang H. Diversity analysis of gut microbiota between healthy controls and those with atopic dermatitis in a Chinese population. J Dermatol. 2021;48:158–67. 32860635 10.1111/1346-8138.15530

[57] Liu X Cai M Chen M. Alterations in gut microbiome associated with severity of atopic dermatitis in infants. Australas J Dermatol. 2024;65:328–36. 38419203 10.1111/ajd.14237

[58] Wang Y Choy CT Lin Y. Effect of a novel E3 probiotics formula on the gut microbiome in atopic dermatitis patients: a pilot study. Biomedicines. 2022;10:2904. 36428472 10.3390/biomedicines10112904 PMC9687608

[59] Hong R-P Hou Y-Y Xu X-J. The difference of gut microbiota and their correlations with urinary organic acids between autistic children with and without atopic dermatitis. Front Cell Infect Microbiol. 2022;12:886196. 35800387 10.3389/fcimb.2022.886196 PMC9253573

[60] Fiocchi A Burks W Bahna SL. Clinical Use of Probiotics in Pediatric Allergy (CUPPA): a world allergy organization position paper. World Allergy Organ J. 2012;5:148–67. 23282383 10.1097/WOX.0b013e3182784ee0 PMC3651185

[61] Kim JY Kwon JH Ahn SH. Effect of probiotic mix (*Bifidobacterium bifidum*, *Bifidobacterium lactis*, *Lactobacillus acidophilus*) in the primary prevention of eczema: a double-blind, randomized, placebo-controlled trial. Pediatr Allergy Immunol. 2010;21(2 Pt 2):e386–93. 19840300 10.1111/j.1399-3038.2009.00958.x

[62] Licari A Marseglia A Castellazzi AM. Atopic dermatitis: is there a role for probiotics. J Biol Regul Homeost Agents. 2015;29(2 Suppl 1):18–24. 26634583

[63] Osborn DA Sinn JK. Probiotics in infants for prevention of allergic disease and food hypersensitivity. Cochrane Database Syst Rev. 2007:CD006475. 17943911 10.1002/14651858.CD006474.pub2

[64] Pan S-J Kuo C-H Lam K-P Chu Y-T Wang W-L Hung C-H. Probiotics and allergy in children—an update review. Pediatr Allergy Immunol. 2010;21(4 Pt 2):e659–66. 20659267 10.1111/j.1399-3038.2010.01061.x

