# Supplementary material for: Exploring the causal relationship between gut microbiota and atopic dermatitis: A Mendelian randomization study
Source: Medicine (Baltimore). 2024 Dec 27;103(52):e40193. doi: 10.1097/MD.0000000000040193 (PMC11688022; doi:10.1097/MD.0000000000040193)
Supplement: Supplementary file 1 [file medi-103-e40193-s001.docx]

Supplementary Files


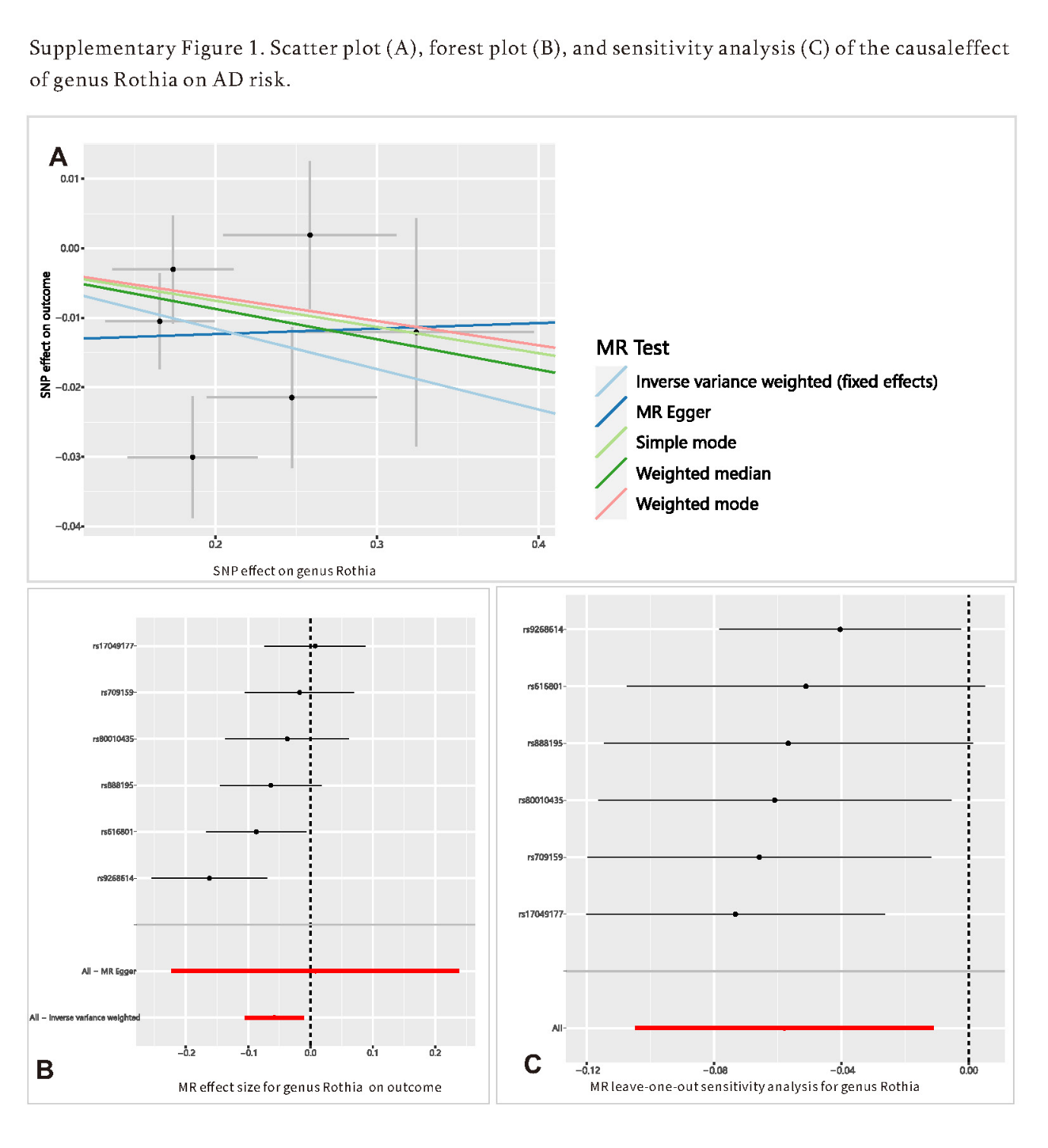


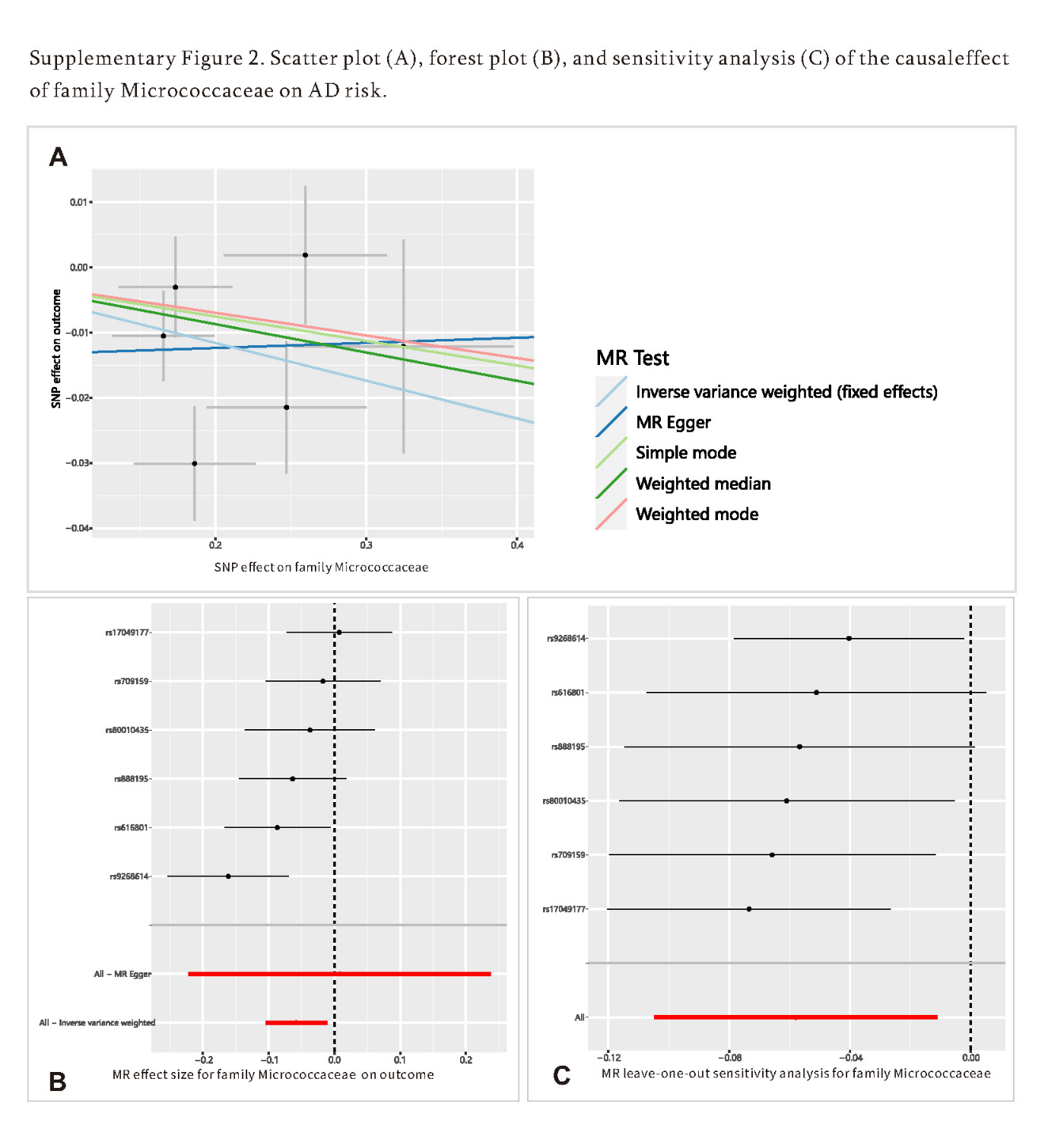


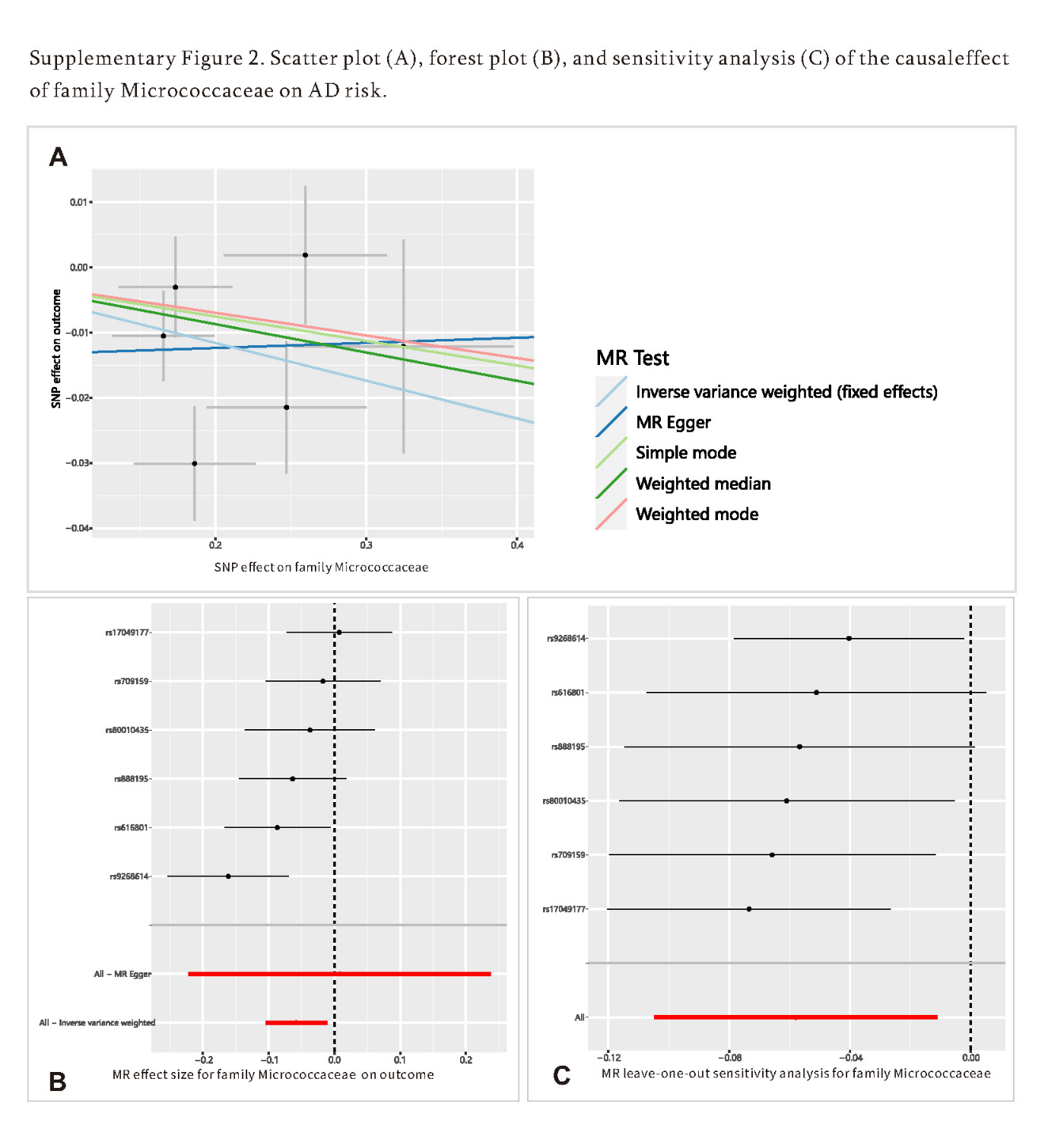


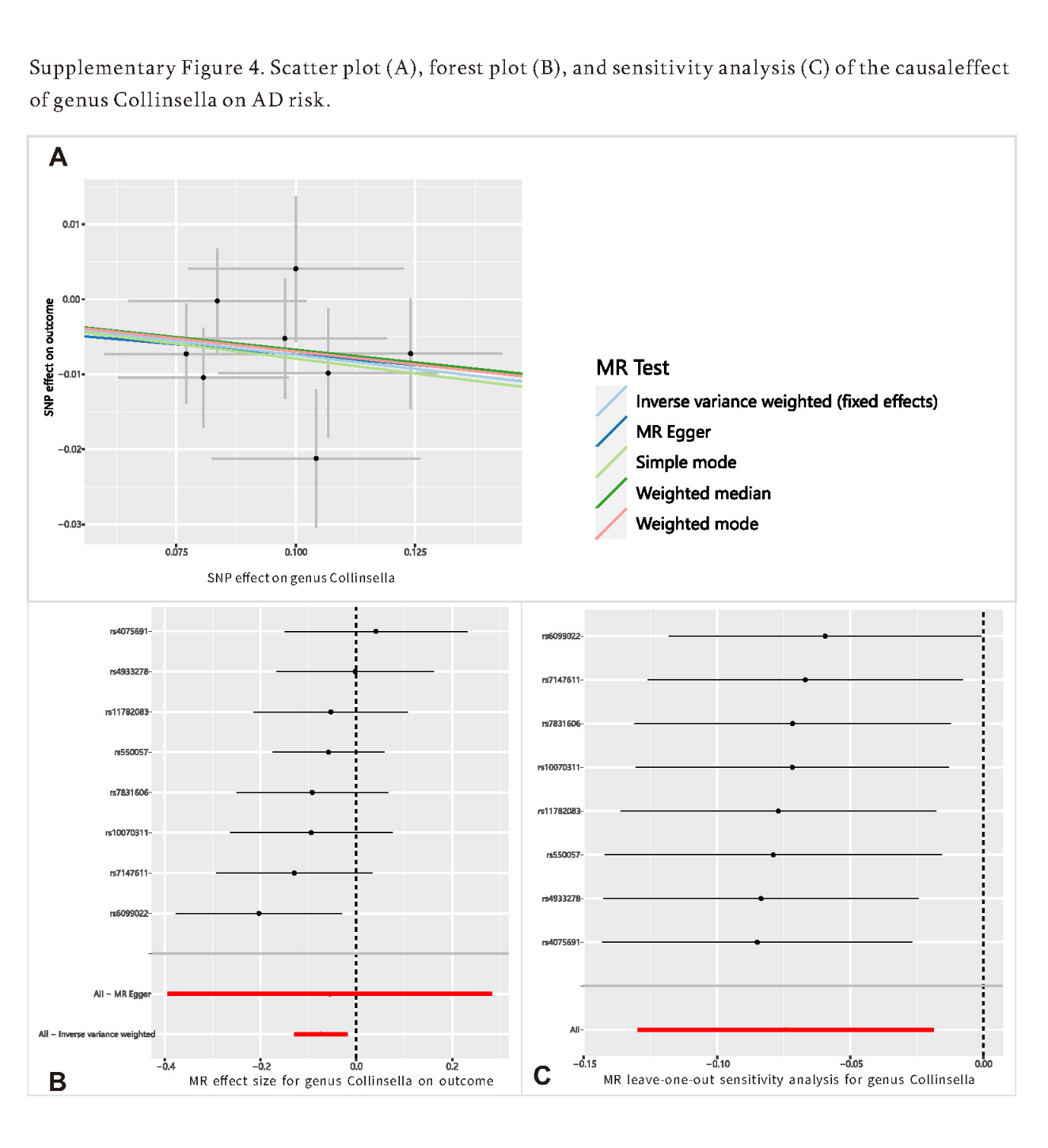


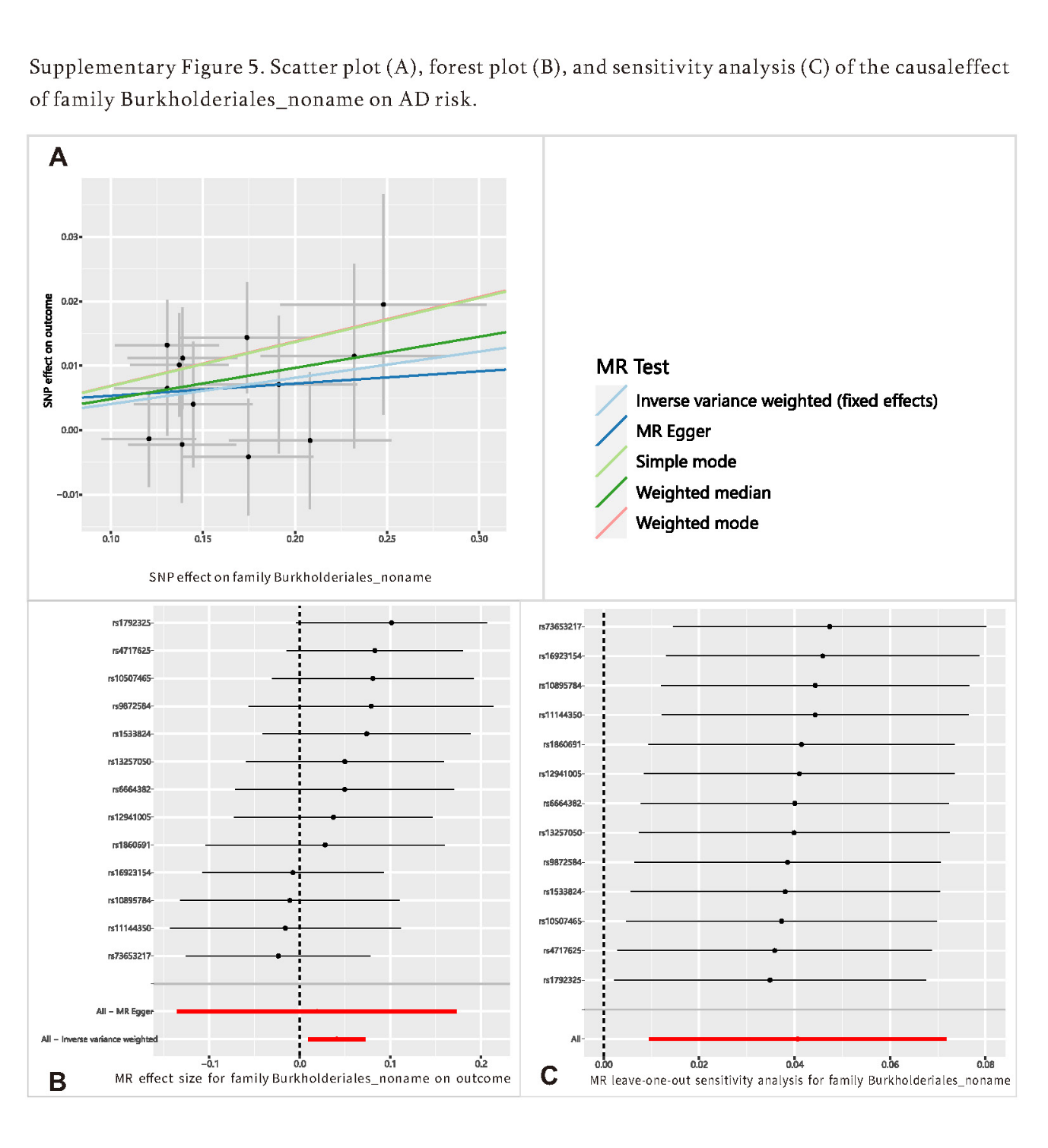


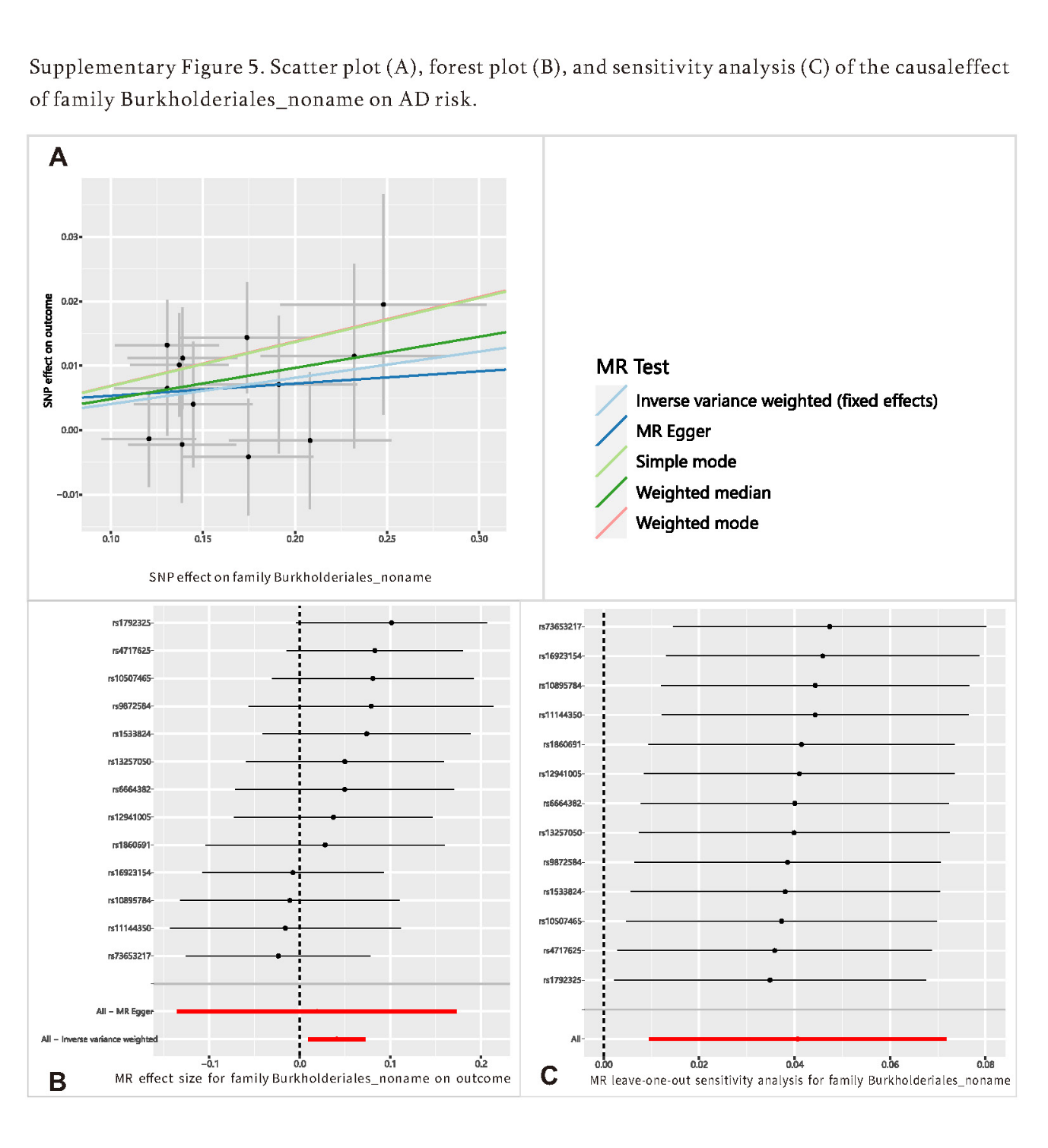


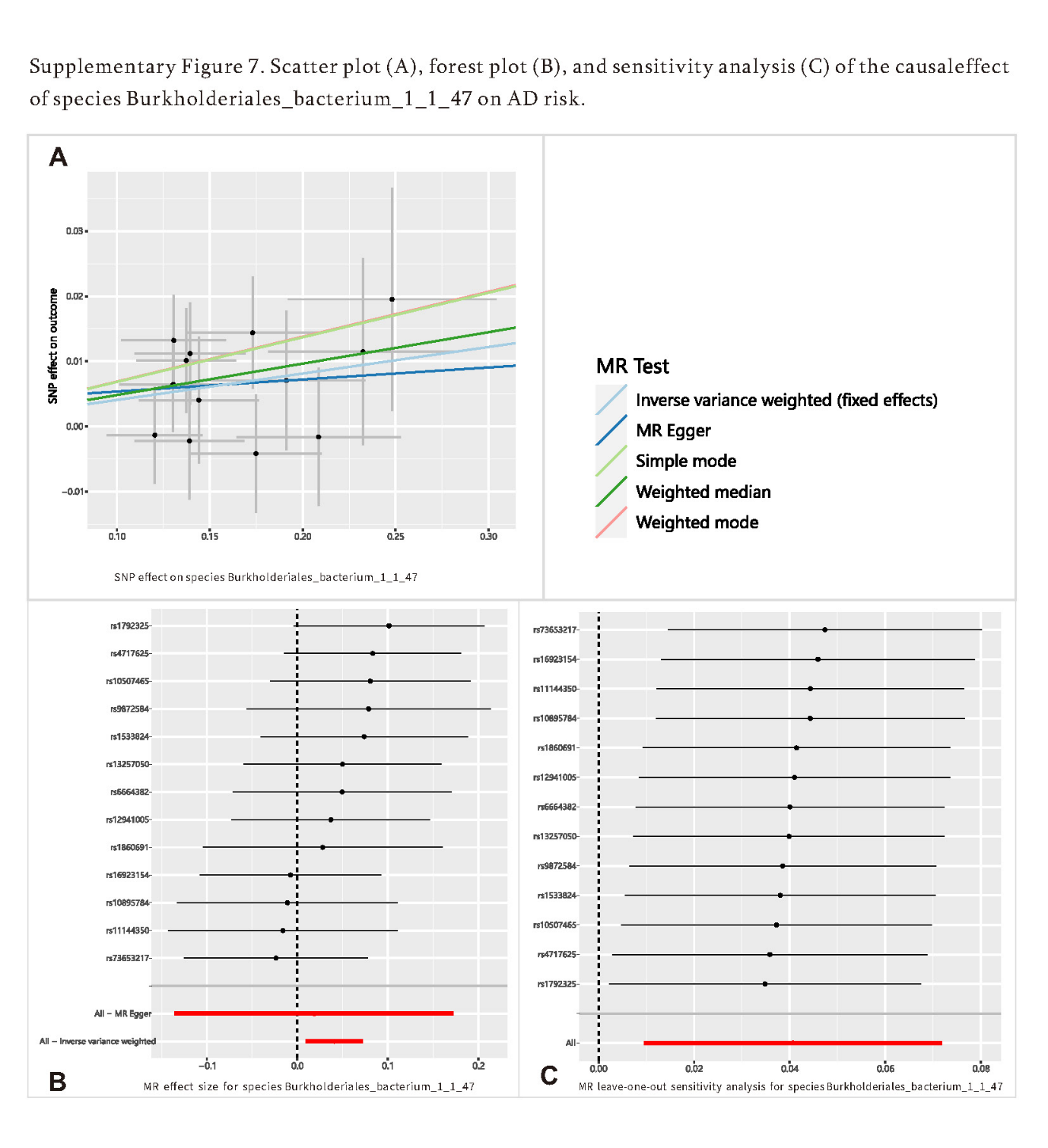


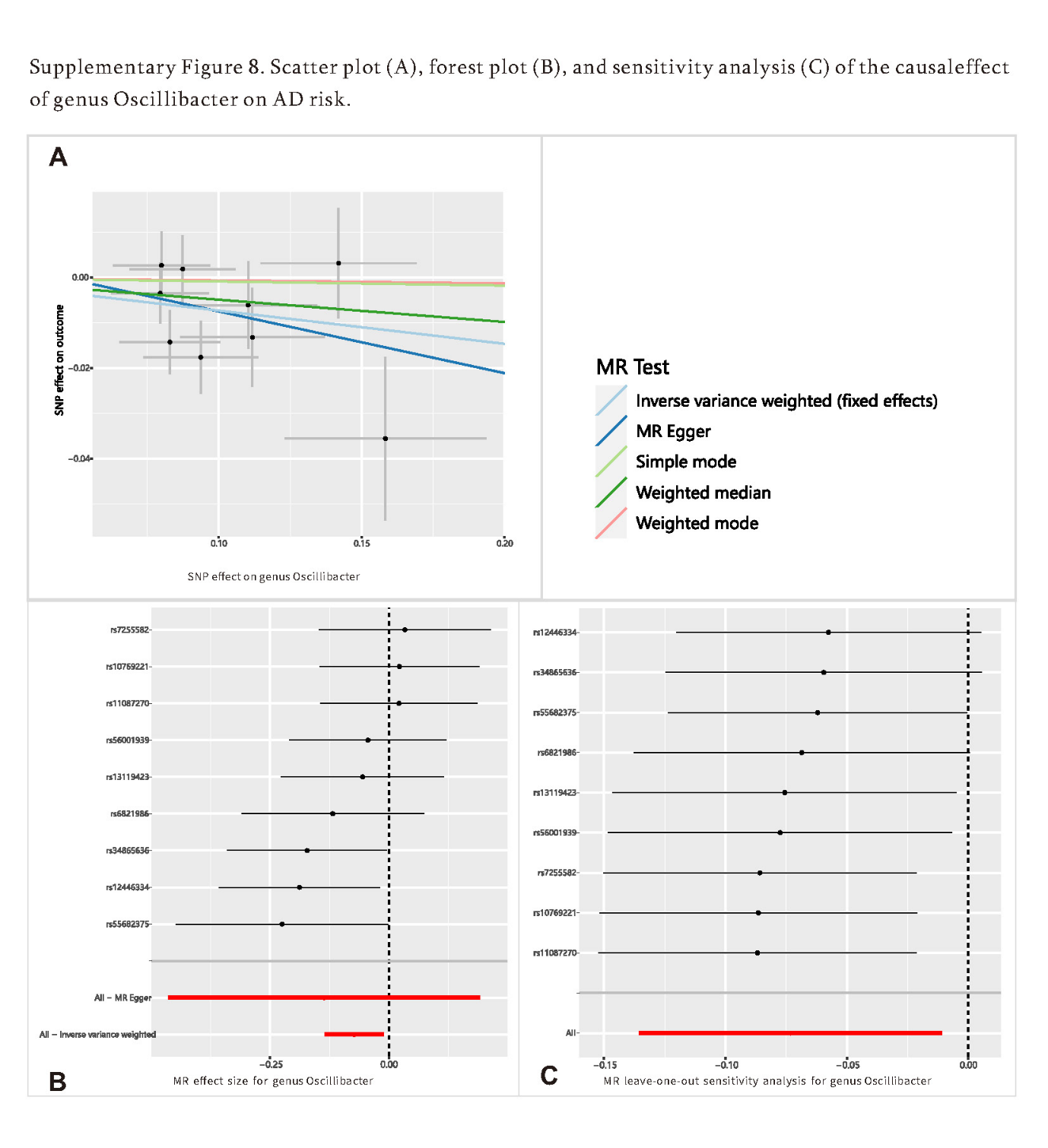


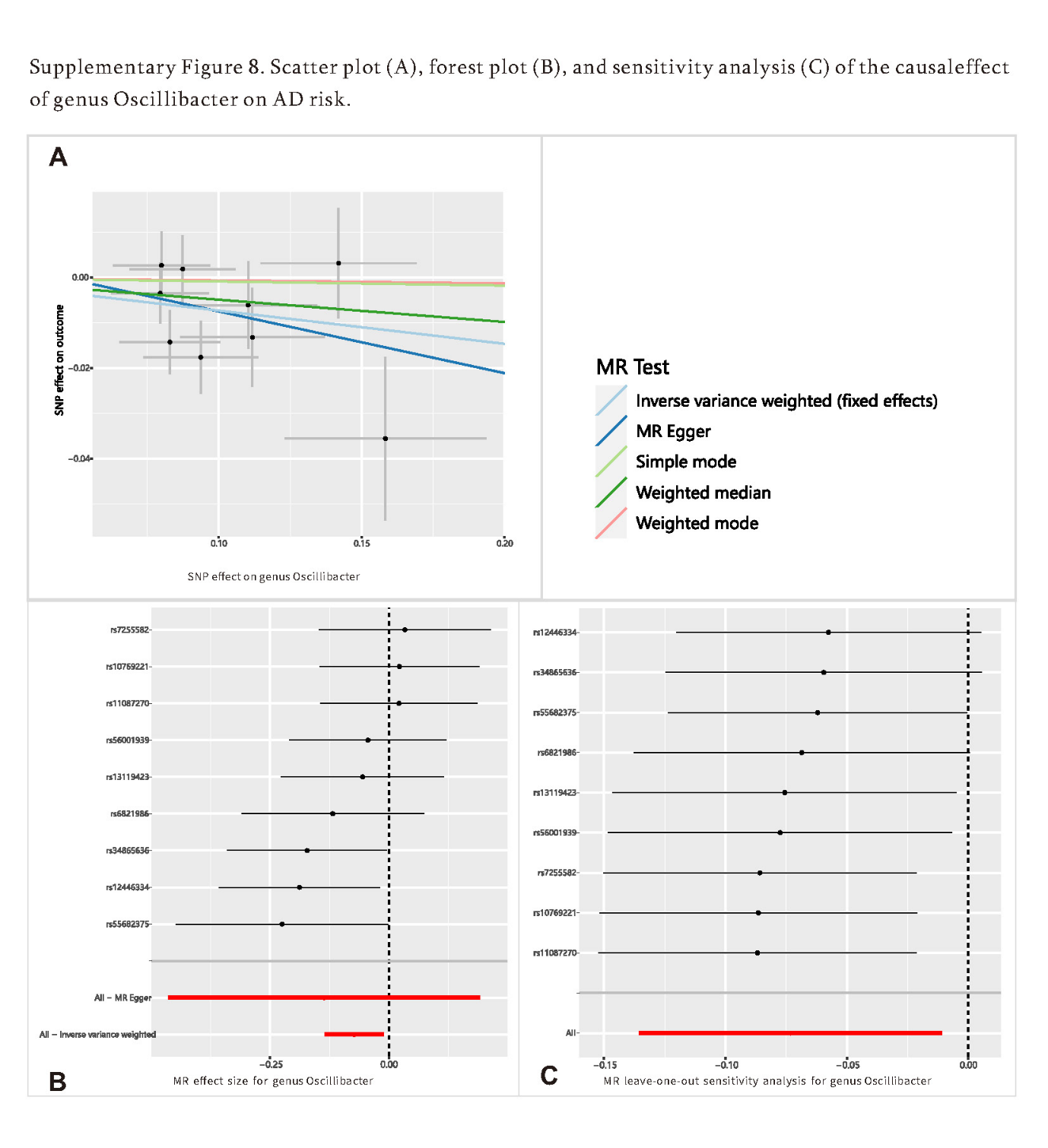


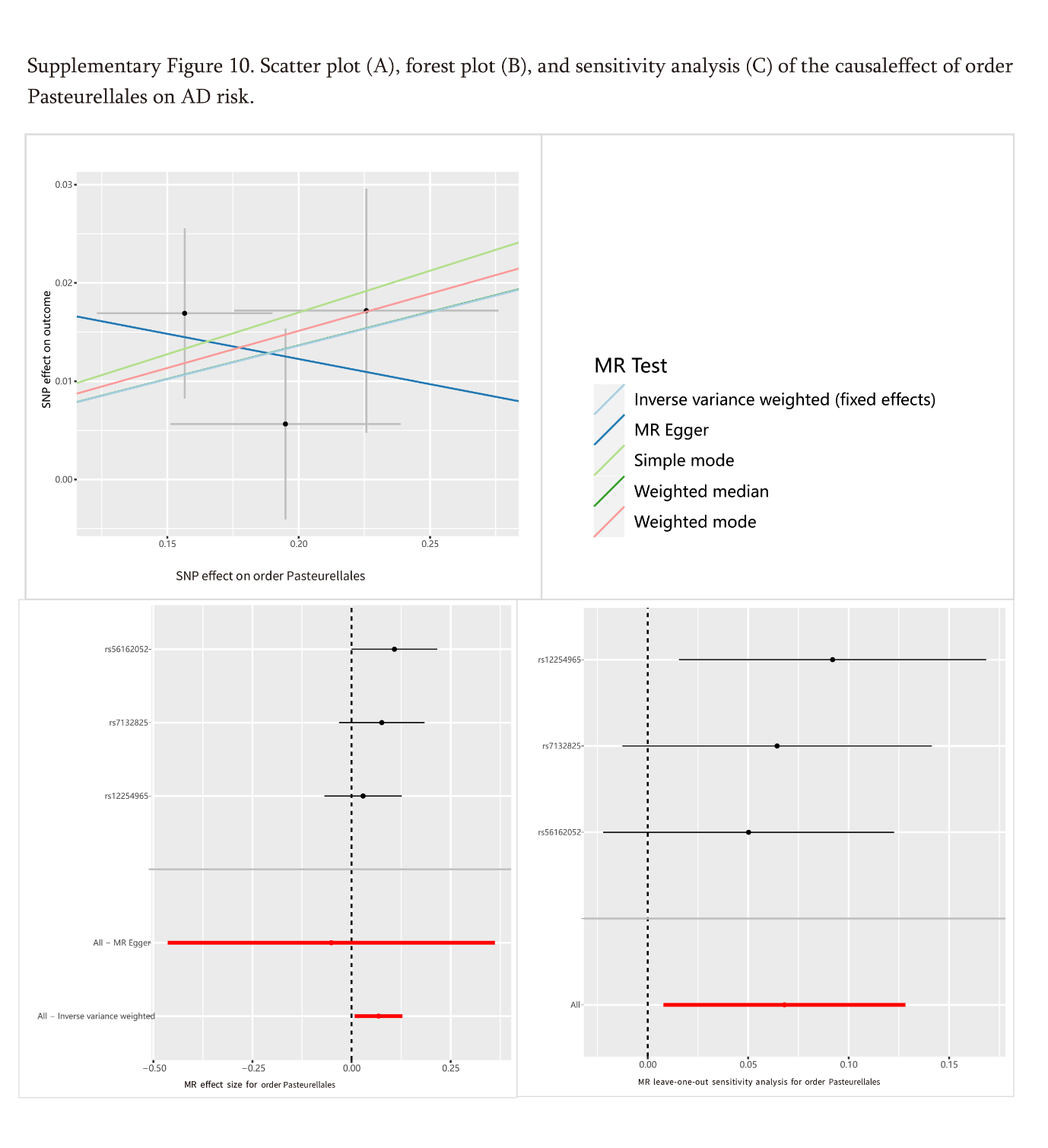


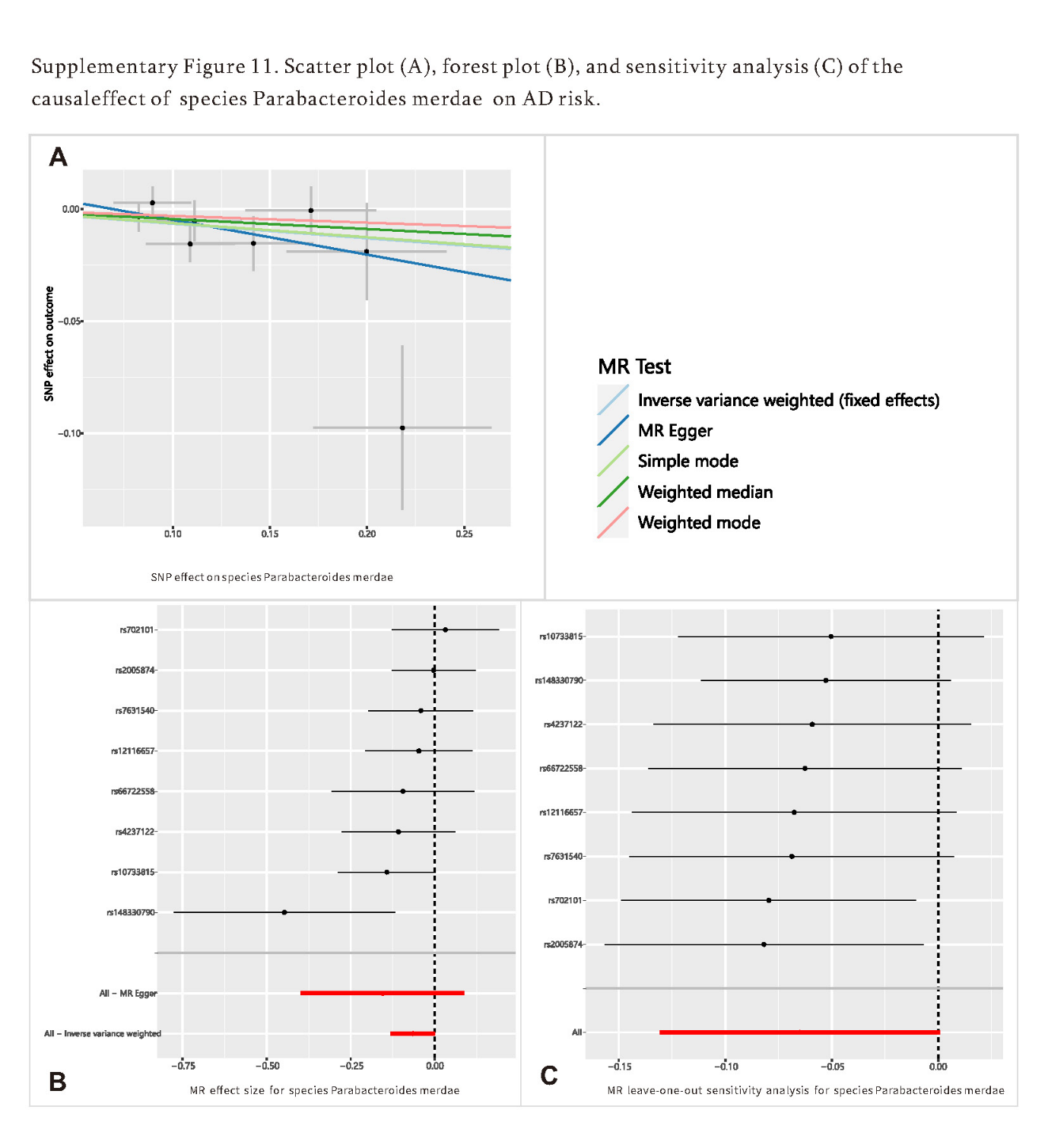


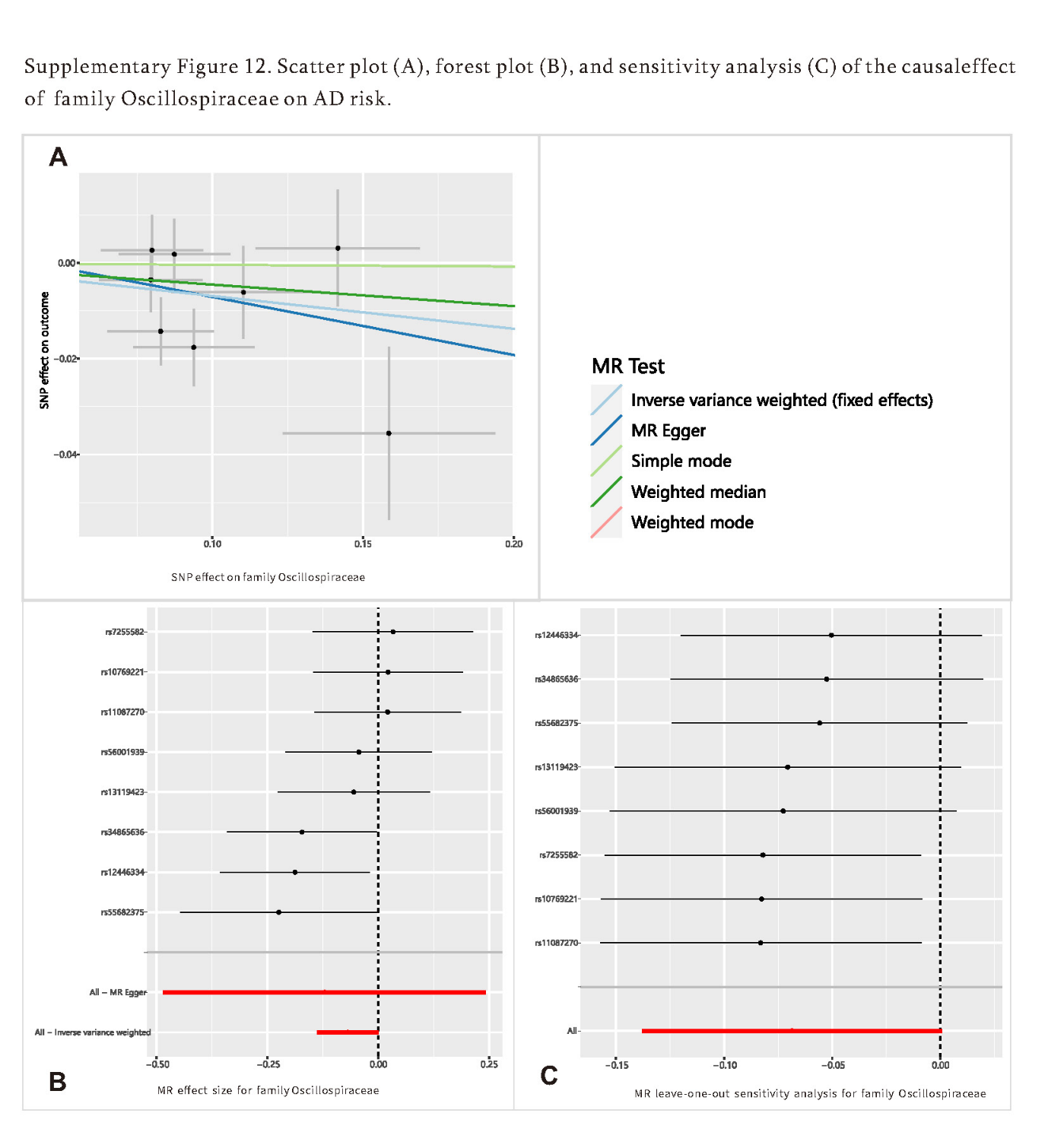


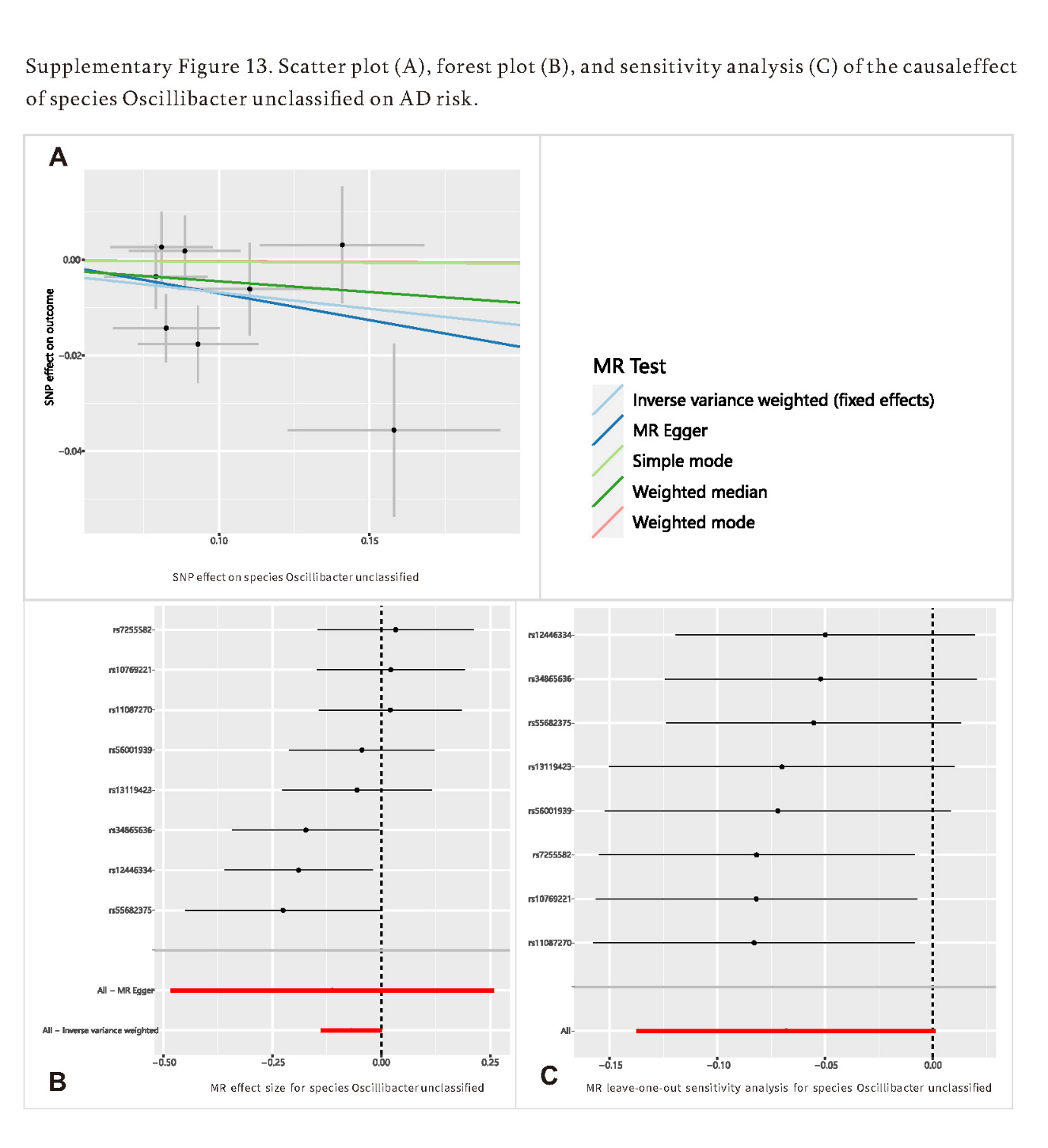


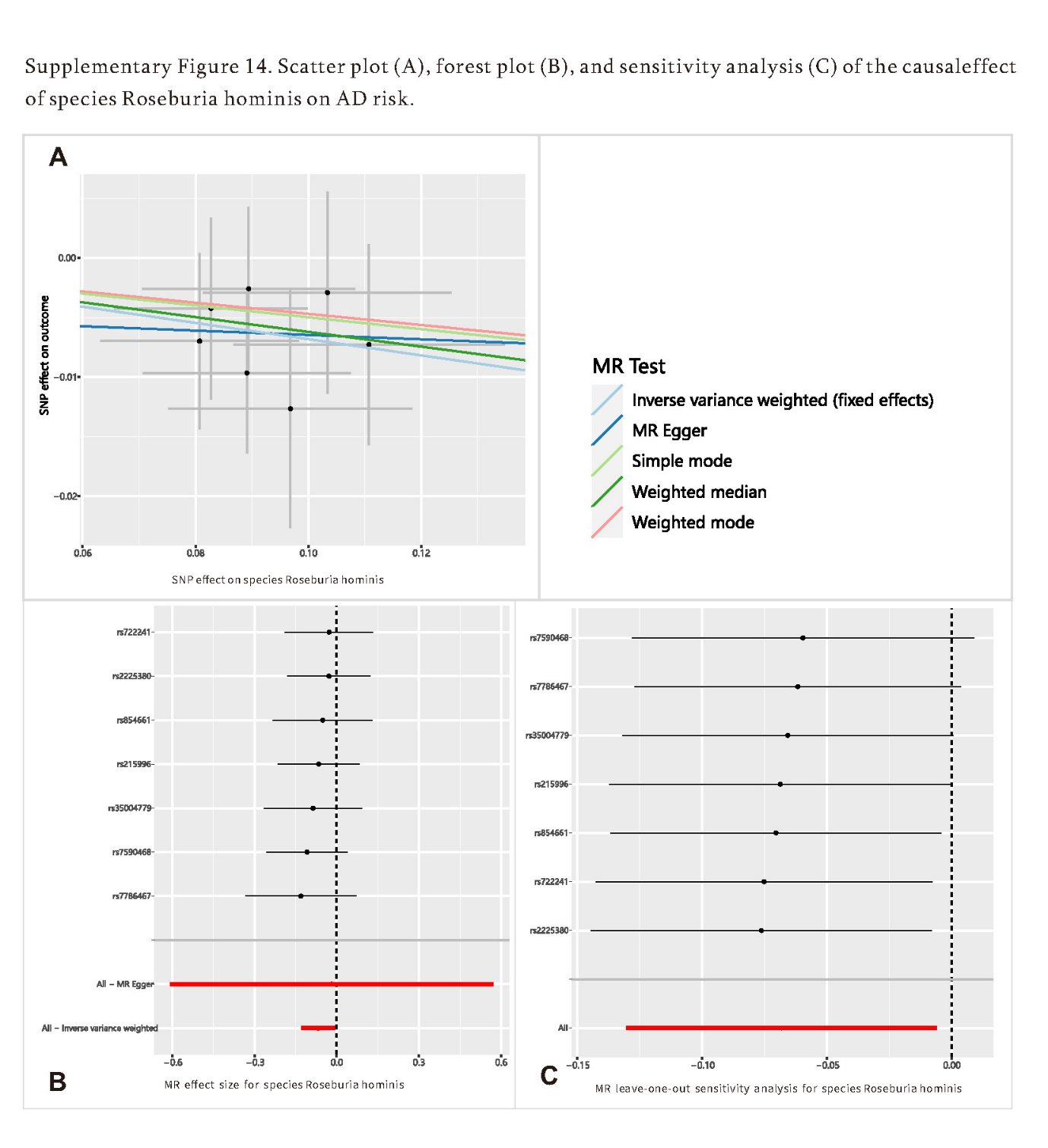


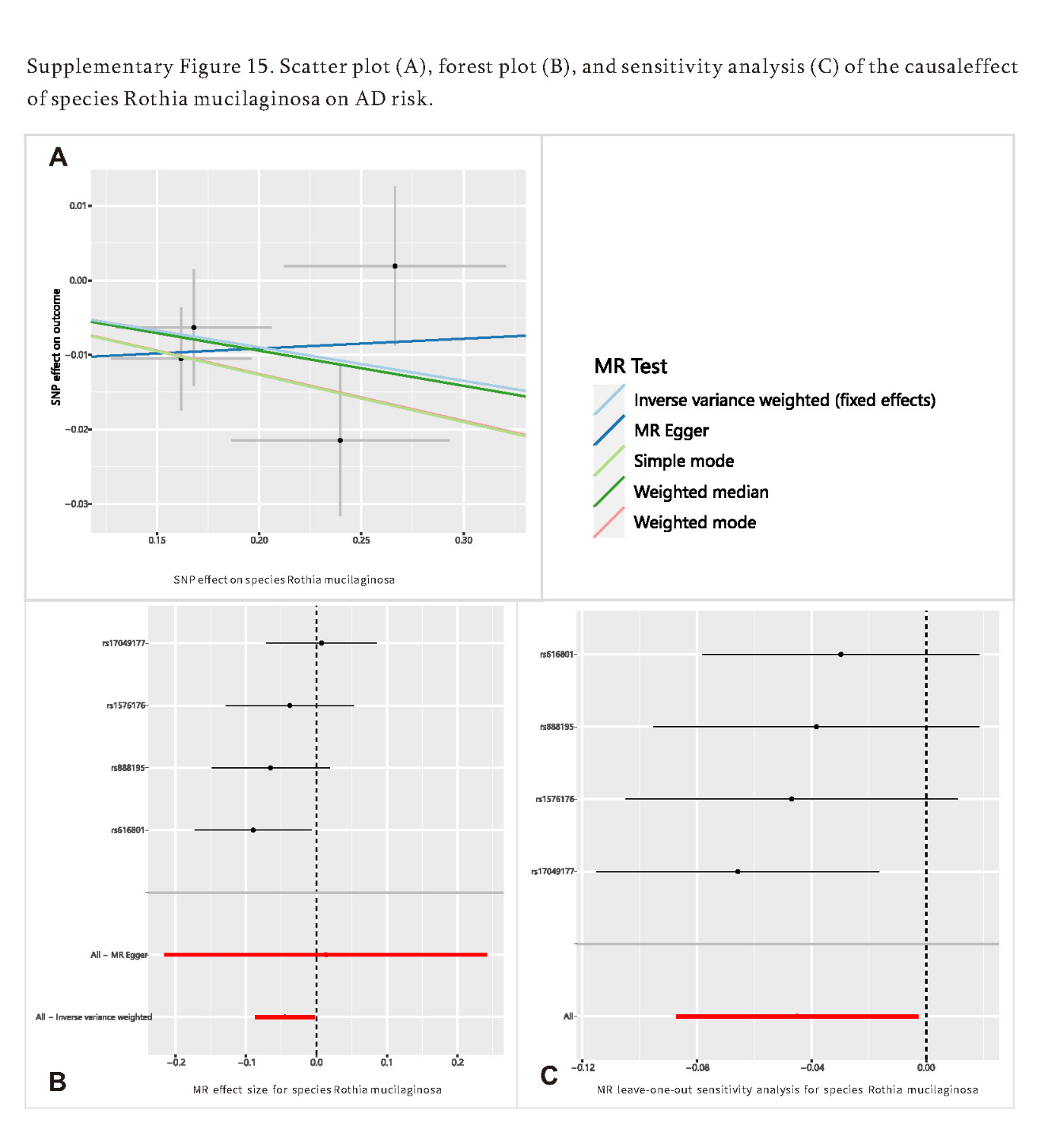


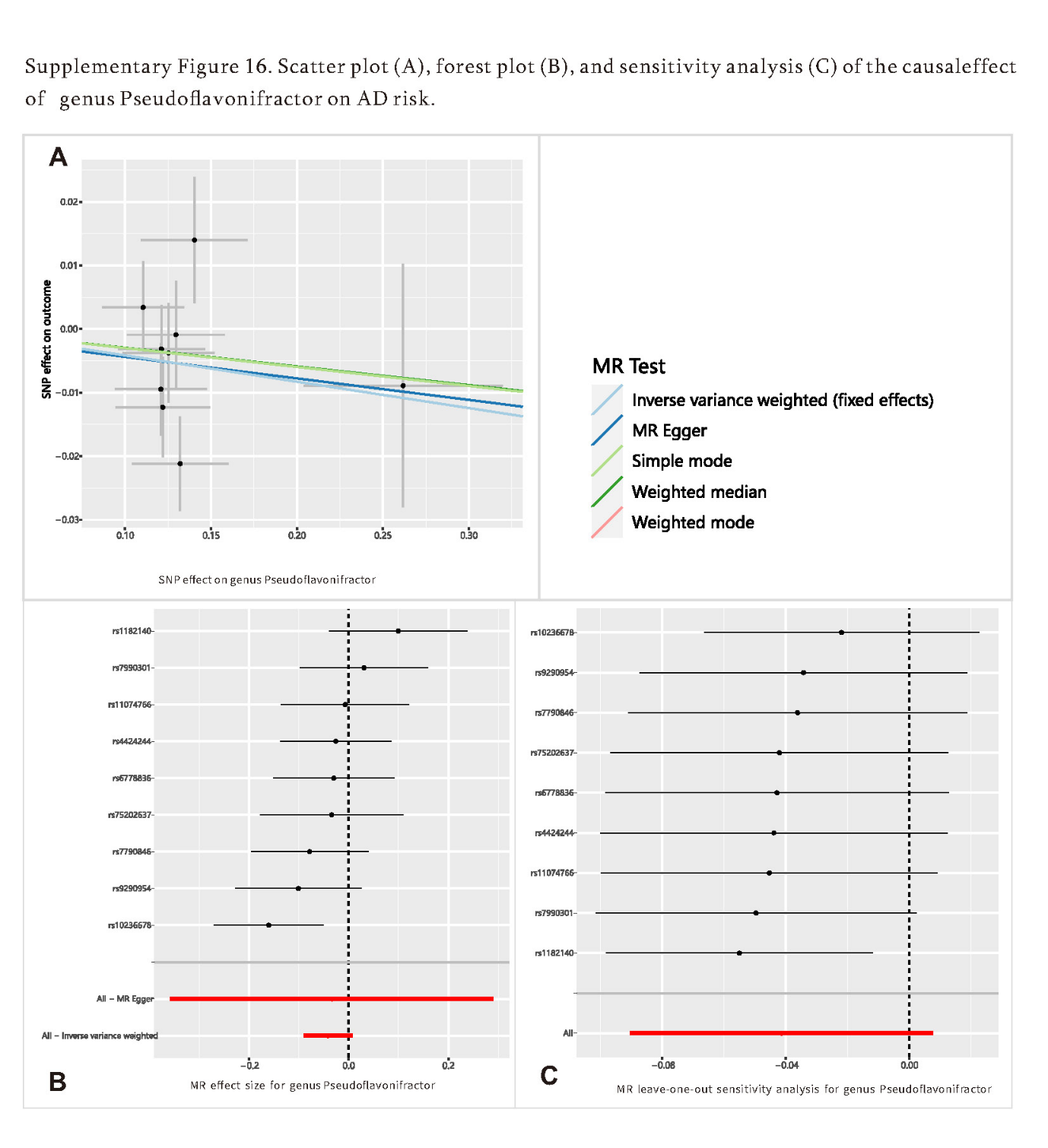


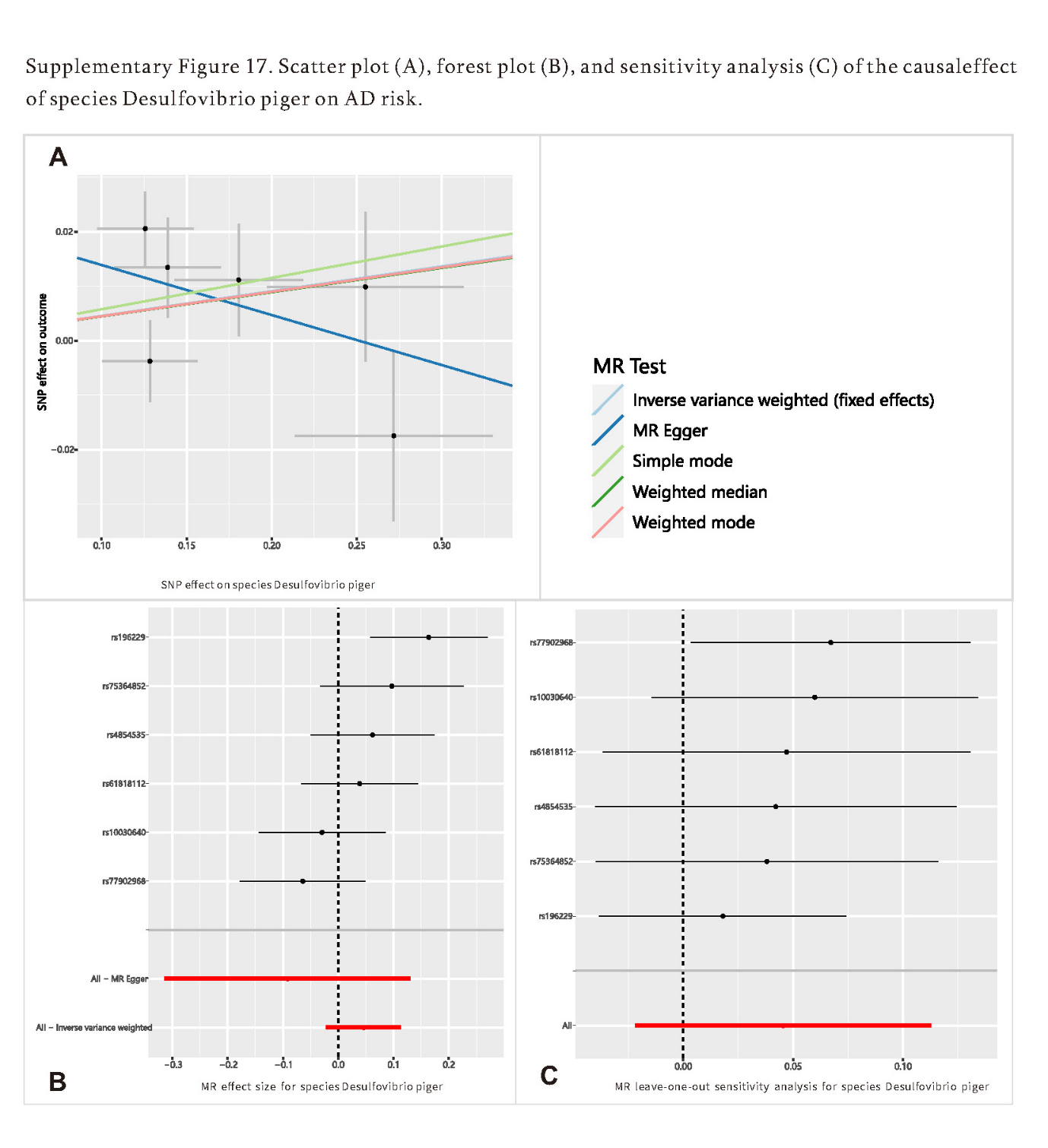


**Supplementary Table 1.** Features to be considered in the diagnosis of patients with atopic dermatitis^[10]^

| **ESSENTIAL FEATURES**—Must be present:  •Pruritus  •Eczema (acute, subacute, chronic)  □ Typical morphology and age-specific patterns*  □ Chronic or relapsing history  *Patterns include:  1. Facial, neck, and extensor involvement in infants and children  2. Current or previous flexural lesions in any age group  3. Sparing of the groin and axillary regions |
| --- |
| **IMPORTANT FEATURES**—Seen in most cases, adding support to the diagnosis:  •Early age of onset  •Atopy  □ Personal and/or family history  □ Immunoglobulin E reactivity  •Xerosis |
| **ASSOCIATED FEATURES**—These clinical associations help to suggest the diagnosis of atopic dermatitis but are too nonspecific to be used for defining or detecting atopic dermatitis for research and epidemiologic studies:  •Atypical vascular responses (eg, facial pallor, white dermographism, delayed blanch response)  •Keratosis pilaris/pityriasis alba/hyperlinear palms/ichthyosis  •Ocular/periorbital changes  •Other regional findings (eg, perioral changes/periauricular lesions)  •Perifollicular accentuation/lichenification/prurigo lesions |
| **EXCLUSIONARY CONDITIONS**—It should be noted that a diagnosis of atopic dermatitis depends on excluding conditions, such as:  •Scabies  •Seborrheic dermatitis  •Contact dermatitis (irritant or allergic)  •Ichthyoses  •Cutaneous T-cell lymphoma  •Psoriasis  •Photosensitivity dermatoses  •Immune deficiency diseases  •Erythroderma of other causes |

**Supplementary Table 2.** SNPs were used as instrumental variables from gut microbiome and AD GWASs (P <1 ×10^-5^).

| **Exposure** | **N** | **SNP** | **Chr** | **Position** | **Beta.exposure** | **SE.exposure** | **Pval.exposure** | **Beta.outcome** | **SE.outcome** | **Pval.outcome** | **F** |
| --- | --- | --- | --- | --- | --- | --- | --- | --- | --- | --- | --- |
| Genus Rothia  (ebi-a-GSCT90027687) | 1 | rs17049177 | 2 | 129915467 | -0.258588319 | 0.053506524 | 1.34601E-06 | -0.001911826 | 0.01063343 | 0.857277 | 23.35027836 |
|  | 2 | rs616801 | 1 | 95011698 | 0.247294645 | 0.052560736 | 2.53952E-06 | -0.021469836 | 0.010128841 | 0.034051 | 22.13065577 |
|  | 3 | rs709159 | 3 | 12481203 | -0.173838186 | 0.037351545 | 3.25384E-06 | 0.003032398 | 0.00773475 | 0.695023 | 21.65513778 |
|  | 4 | rs80010435 | 9 | 140767792 | -0.324346887 | 0.072607333 | 7.92735E-06 | 0.012084685 | 0.016381766 | 0.460699 | 19.95015305 |
|  | 5 | rs888195 | 12 | 2834412 | 0.165637964 | 0.033732316 | 9.09073E-07 | -0.010523175 | 0.006895762 | 0.127004 | 24.10544632 |
|  | 6 | rs9268614 | 6 | 32402778 | -0.185915133 | 0.040120478 | 3.58804E-06 | 0.030074203 | 0.008751446 | 0.000593 | 21.46767605 |
| Family Micrococcaceae  (ebi-a-GSCT90027661) | 1 | rs17049177 | 2 | 129915467 | -0.259547835 | 0.05389552 | 1.46641E-06 | -0.001911826 | 0.01063343 | 0.857277 | 23.18554291 |
|  | 2 | rs616801 | 1 | 95011698 | 0.247127192 | 0.05294285 | 3.04427E-06 | -0.021469836 | 0.010128841 | 0.034051 | 21.78282093 |
|  | 3 | rs709159 | 3 | 12481203 | -0.1735348 | 0.03762309 | 3.97942E-06 | 0.003032398 | 0.00773475 | 0.695023 | 21.26923844 |
|  | 4 | rs80010435 | 9 | 140767792 | -0.324558576 | 0.07313519 | 9.08874E-06 | 0.012084685 | 0.016381766 | 0.460699 | 19.68888562 |
|  | 5 | rs888195 | 12 | 2834412 | 0.165584929 | 0.03397755 | 1.09715E-06 | -0.010523175 | 0.006895762 | 0.127004 | 23.7435259 |
|  | 6 | rs9268614 | 6 | 32402778 | -0.186288991 | 0.04043215 | 4.03198E-06 | 0.030074203 | 0.008751446 | 0.000593 | 21.24408948 |
| Order Actinomycetales  (ebi-a-GSCT90027735) | 1 | rs13236023 | 7 | 21591664 | 0.136666794 | 0.030598405 | 7.95216E-06 | -0.005509148 | 0.007271336 | 0.448675 | 19.94417911 |
|  | 2 | rs7334276 | 13 | 43399563 | 0.196265966 | 0.042846528 | 4.6348E-06 | -0.024701588 | 0.009487235 | 0.00924 | 20.97714857 |
|  | 3 | rs888195 | 12 | 2834412 | 0.159693567 | 0.03190596 | 5.58235E-07 | -0.010523175 | 0.006895762 | 0.127004 | 25.0448796 |
| Genus Collinsella  (ebi-a-GSCT90027690) | 1 | rs10070311 | 5 | 79570013 | 0.077074352 | 0.017168111 | 7.14273E-06 | -0.00727238 | 0.006654946 | 0.27446 | 20.14941568 |
|  | 2 | rs11782083 | 8 | 20549398 | -0.097709073 | 0.02131237 | 4.54817E-06 | 0.005218361 | 0.008015858 | 0.515003 | 21.01328955 |
|  | 3 | rs4075691 | 15 | 92002949 | -0.100005144 | 0.022493262 | 8.74821E-06 | -0.004080313 | 0.009716224 | 0.674575 | 19.76184631 |
|  | 4 | rs4933278 | 10 | 84743037 | -0.083550136 | 0.018618472 | 7.20685E-06 | 0.000213977 | 0.006993707 | 0.975562 | 20.13232821 |
|  | 5 | rs550057 | 9 | 136146597 | 0.124063271 | 0.01915381 | 9.34335E-11 | -0.007241154 | 0.007338564 | 0.323762 | 41.94341872 |
|  | 6 | rs6099022 | 20 | 54747624 | -0.104290989 | 0.021849987 | 1.81461E-06 | 0.021210459 | 0.009227169 | 0.021551 | 22.77607786 |
|  | 7 | rs7147611 | 14 | 95024931 | -0.080652431 | 0.017853301 | 6.25717E-06 | 0.010451195 | 0.006680826 | 0.117751 | 20.40260359 |
|  | 8 | rs7831606 | 8 | 128455694 | -0.106800412 | 0.022999528 | 3.42404E-06 | 0.009834483 | 0.008610003 | 0.253348 | 21.55736947 |
| Family Burkholderiales_noname  (ebi-a-GSCT90027680) | 1 | rs10507465 | 13 | 38463552 | -0.139036721 | 0.029764189 | 2.99335E-06 | -0.011204537 | 0.007875801 | 0.154828 | 21.81517288 |
|  | 2 | rs10895784 | 11 | 105046839 | 0.120713508 | 0.025717285 | 2.68089E-06 | -0.001330885 | 0.007467336 | 0.858508 | 22.02668976 |
|  | 3 | rs11144350 | 9 | 77862854 | -0.138724816 | 0.029365015 | 2.31077E-06 | 0.002229513 | 0.008996886 | 0.804259 | 22.31185036 |
|  | 4 | rs12941005 | 17 | 29755009 | -0.191247211 | 0.042409204 | 6.49596E-06 | -0.00707094 | 0.010682827 | 0.508031 | 20.33095175 |
|  | 5 | rs13257050 | 8 | 19029859 | 0.130578186 | 0.02876455 | 5.63738E-06 | 0.00648294 | 0.007271227 | 0.372674 | 20.60221032 |
|  | 6 | rs1533824 | 11 | 214169 | 0.137172858 | 0.026635569 | 2.60503E-07 | 0.010118634 | 0.008018745 | 0.206991 | 26.51552093 |
|  | 7 | rs16923154 | 12 | 20885931 | 0.208220802 | 0.044087866 | 2.32551E-06 | -0.00159427 | 0.01061007 | 0.880516 | 22.29963813 |
|  | 8 | rs1792325 | 11 | 71113304 | -0.130590496 | 0.028212798 | 3.67847E-06 | -0.013206827 | 0.007002905 | 0.059316 | 21.41995595 |
|  | 9 | rs1860691 | 7 | 52336084 | 0.144646686 | 0.032212079 | 7.10733E-06 | 0.004030865 | 0.009737191 | 0.678949 | 20.15891728 |
|  | 10 | rs4717625 | 7 | 71508645 | 0.17390615 | 0.035894726 | 1.26679E-06 | 0.014385037 | 0.008607338 | 0.094666 | 23.46693255 |
|  | 11 | rs6664382 | 1 | 76089534 | 0.232223748 | 0.050739583 | 4.72195E-06 | 0.011491717 | 0.014306861 | 0.421829 | 20.94147205 |
|  | 12 | rs73653217 | 9 | 79442588 | -0.174748492 | 0.035174459 | 6.76293E-07 | 0.004146392 | 0.00906417 | 0.647398 | 24.67514751 |
|  | 13 | rs9872584 | 3 | 178567864 | -0.248008458 | 0.055944644 | 9.28858E-06 | -0.019517228 | 0.017080774 | 0.253171 | 19.6473371 |
| Genus Burkholderiales_noname  (ebi-a-GSCT90027725) | 1 | rs10507465 | 13 | 38463552 | -0.138845622 | 0.029775431 | 3.11491E-06 | -0.011204537 | 0.007875801 | 0.154828 | 21.73882181 |
|  | 2 | rs10895784 | 11 | 105046839 | 0.12084943 | 0.025731426 | 2.64567E-06 | -0.001330885 | 0.007467336 | 0.858508 | 22.0520636 |
|  | 3 | rs11144350 | 9 | 77862854 | -0.138537498 | 0.02937266 | 2.39882E-06 | 0.002229513 | 0.008996886 | 0.804259 | 22.24005538 |
|  | 4 | rs12941005 | 17 | 29755009 | -0.191666382 | 0.042436497 | 6.28565E-06 | -0.00707094 | 0.010682827 | 0.508031 | 20.39391369 |
|  | 5 | rs13257050 | 8 | 19029859 | 0.130366303 | 0.028776268 | 5.88886E-06 | 0.00648294 | 0.007271227 | 0.372674 | 20.5186832 |
|  | 6 | rs1533824 | 11 | 214169 | 0.137378622 | 0.026645439 | 2.52547E-07 | 0.010118634 | 0.008018745 | 0.206991 | 26.5754284 |
|  | 7 | rs16923154 | 12 | 20885931 | 0.208234256 | 0.044093878 | 2.32939E-06 | -0.00159427 | 0.01061007 | 0.880516 | 22.29643849 |
|  | 8 | rs1792325 | 11 | 71113304 | -0.130505554 | 0.028235016 | 3.79843E-06 | -0.013206827 | 0.007002905 | 0.059316 | 21.35844586 |
|  | 9 | rs1860691 | 7 | 52336084 | 0.144272372 | 0.032233595 | 7.6112E-06 | 0.004030865 | 0.009737191 | 0.678949 | 20.02795363 |
|  | 10 | rs4717625 | 7 | 71508645 | 0.173645495 | 0.03591153 | 1.32899E-06 | 0.014385037 | 0.008607338 | 0.094666 | 23.37474952 |
|  | 11 | rs6664382 | 1 | 76089534 | 0.23183956 | 0.050745157 | 4.90751E-06 | 0.011491717 | 0.014306861 | 0.421829 | 20.86765399 |
|  | 12 | rs73653217 | 9 | 79442588 | -0.174787635 | 0.03519085 | 6.80496E-07 | 0.004146392 | 0.00906417 | 0.647398 | 24.66321171 |
|  | 13 | rs9872584 | 3 | 178567864 | -0.247588593 | 0.055952726 | 9.646E-06 | -0.019517228 | 0.017080774 | 0.253171 | 19.57521292 |
| Species Burkholderiales_bacterium_1_1_47  (ebi-a-GSCT90027809) | 1 | rs10507465 | 13 | 38463552 | -0.139392017 | 0.029775847 | 2.84952E-06 | -0.011204537 | 0.007875801 | 0.154828 | 21.90964306 |
|  | 2 | rs10895784 | 11 | 105046839 | 0.120402237 | 0.025731785 | 2.88102E-06 | -0.001330885 | 0.007467336 | 0.858508 | 21.88855075 |
|  | 3 | rs11144350 | 9 | 77862854 | -0.13907398 | 0.02937307 | 2.19331E-06 | 0.002229513 | 0.008996886 | 0.804259 | 22.41201087 |
|  | 4 | rs12941005 | 17 | 29755009 | -0.191230381 | 0.042437089 | 6.59962E-06 | -0.00707094 | 0.010682827 | 0.508031 | 20.30066861 |
|  | 5 | rs13257050 | 8 | 19029859 | 0.130267225 | 0.02877667 | 5.98738E-06 | 0.00648294 | 0.007271227 | 0.372674 | 20.48693472 |
|  | 6 | rs1533824 | 11 | 214169 | 0.137332021 | 0.026645812 | 2.55013E-07 | 0.010118634 | 0.008018745 | 0.206991 | 26.55666006 |
|  | 7 | rs16923154 | 12 | 20885931 | 0.208534234 | 0.044094494 | 2.25342E-06 | -0.00159427 | 0.01061007 | 0.880516 | 22.36009986 |
|  | 8 | rs1792325 | 11 | 71113304 | -0.130563026 | 0.02823541 | 3.7625E-06 | -0.013206827 | 0.007002905 | 0.059316 | 21.37666482 |
|  | 9 | rs1860691 | 7 | 52336084 | 0.144091935 | 0.032234045 | 7.81544E-06 | 0.004030865 | 0.009737191 | 0.678949 | 19.97733036 |
|  | 10 | rs4717625 | 7 | 71508645 | 0.173022567 | 0.035912031 | 1.45037E-06 | 0.014385037 | 0.008607338 | 0.094666 | 23.20669538 |
|  | 11 | rs6664382 | 1 | 76089534 | 0.232399814 | 0.050745865 | 4.65688E-06 | 0.011491717 | 0.014306861 | 0.421829 | 20.96804611 |
|  | 12 | rs73653217 | 9 | 79442588 | -0.174870074 | 0.035191341 | 6.72567E-07 | 0.004146392 | 0.00906417 | 0.647398 | 24.68579283 |
|  | 13 | rs9872584 | 3 | 178567864 | -0.248138963 | 0.055953508 | 9.21857E-06 | -0.019517228 | 0.017080774 | 0.253171 | 19.66178876 |
| Genus Oscillibacter  (ebi-a-GSCT90027714) | 1 | rs10769221 | 11 | 5806426 | 0.141916501 | 0.027228668 | 1.8679E-07 | 0.003124115 | 0.012168263 | 0.797368 | 27.15818459 |
|  | 2 | rs11087270 | 20 | 18906606 | 0.08734109 | 0.018519724 | 2.40394E-06 | 0.001850287 | 0.007379044 | 0.802032 | 22.23596319 |
|  | 3 | rs12446334 | 16 | 81302009 | 0.093675539 | 0.020060991 | 3.01879E-06 | -0.017615241 | 0.008086062 | 0.02939 | 21.79893972 |
|  | 4 | rs13119423 | 4 | 27850234 | -0.110310128 | 0.02398116 | 4.22762E-06 | 0.006111288 | 0.009649478 | 0.526542 | 21.15329982 |
|  | 5 | rs34865636 | 19 | 45793832 | -0.082905862 | 0.017673967 | 2.72077E-06 | 0.014257872 | 0.007105204 | 0.044821 | 21.99835065 |
|  | 6 | rs55682375 | 9 | 21808352 | -0.158273517 | 0.035200688 | 6.914E-06 | 0.03555342 | 0.018042109 | 0.048787 | 20.21165804 |
|  | 7 | rs56001939 | 2 | 54363878 | -0.079486561 | 0.017043137 | 3.10354E-06 | 0.003486914 | 0.006705632 | 0.603087 | 21.74583432 |
|  | 8 | rs6821986 | 4 | 181658296 | -0.111752033 | 0.02529687 | 9.97923E-06 | 0.013217266 | 0.010945267 | 0.227205 | 19.51034923 |
|  | 9 | rs7255582 | 19 | 35718076 | -0.079963845 | 0.016918517 | 2.28524E-06 | -0.002682595 | 0.007379552 | 0.716189 | 22.33317637 |
| Family Pasteurellaceae  (ebi-a-GSCT90027685) | 1 | rs12254965 | 10 | 127297038 | -0.19489732 | 0.043794401 | 8.57587E-06 | -0.005648925 | 0.009713713 | 0.560899 | 19.7998677 |
|  | 2 | rs56162052 | 17 | 36205502 | -0.156606686 | 0.033411034 | 2.76868E-06 | -0.01691526 | 0.008656764 | 0.050714 | 21.96486297 |
|  | 3 | rs7132825 | 12 | 1500603 | 0.225739591 | 0.050319879 | 7.25415E-06 | 0.01718548 | 0.012428292 | 0.16673 | 20.11981672 |
| Order Pasteurellales  (ebi-a-GSCT90027746) | 1 | rs12254965 | 10 | 127297038 | -0.194968953 | 0.043794422 | 8.51087E-06 | -0.005648925 | 0.009713713 | 0.560899 | 19.81440557 |
|  | 2 | rs56162052 | 17 | 36205502 | -0.156599959 | 0.03341105 | 2.77143E-06 | -0.01691526 | 0.008656764 | 0.050714 | 21.96295461 |
|  | 3 | rs7132825 | 12 | 1500603 | 0.225746427 | 0.050319904 | 7.24961E-06 | 0.01718548 | 0.012428292 | 0.16673 | 20.12101554 |
| Species Parabacteroides_merdae  (ebi-a-GSCT90027769) | 1 | rs10733815 | 10 | 67316008 | 0.109024761 | 0.022822261 | 1.77821E-06 | -0.015544187 | 0.008066404 | 0.053996 | 22.81501022 |
|  | 2 | rs12116657 | 1 | 63034646 | -0.111274952 | 0.024153615 | 4.08581E-06 | 0.005273073 | 0.009032614 | 0.559366 | 21.21867583 |
|  | 3 | rs148330790 | 22 | 24326693 | 0.218191842 | 0.045711926 | 1.81328E-06 | -0.097538962 | 0.036597859 | 0.007712 | 22.77748257 |
|  | 4 | rs2005874 | 19 | 46810991 | 0.171143673 | 0.033510796 | 3.27105E-07 | -0.000649211 | 0.01084511 | 0.952237 | 26.07593741 |
|  | 5 | rs4237122 | 9 | 133313115 | 0.14155731 | 0.030780303 | 4.2461E-06 | -0.015286242 | 0.012134205 | 0.207734 | 21.14494459 |
|  | 6 | rs66722558 | 6 | 132090239 | 0.199748402 | 0.040947341 | 1.0707E-06 | -0.018943297 | 0.021557405 | 0.379551 | 23.79046669 |
|  | 7 | rs702101 | 5 | 171276393 | 0.089625047 | 0.019843616 | 6.28498E-06 | 0.002812042 | 0.007280224 | 0.699366 | 20.39411708 |
|  | 8 | rs7631540 | 3 | 113830519 | 0.082540202 | 0.018382322 | 7.11582E-06 | -0.003428872 | 0.006541138 | 0.600132 | 20.15663572 |
| Family Oscillospiraceae  (ebi-a-GSCT90027675) | 1 | rs10769221 | 11 | 5806426 | 0.141693698 | 0.027227988 | 1.95073E-07 | 0.003124115 | 0.012168263 | 0.797368 | 27.0743296 |
|  | 2 | rs11087270 | 20 | 18906606 | 0.087381705 | 0.018519261 | 2.3768E-06 | 0.001850287 | 0.007379044 | 0.802032 | 22.25775975 |
|  | 3 | rs12446334 | 16 | 81302009 | 0.093836872 | 0.02006049 | 2.90116E-06 | -0.017615241 | 0.008086062 | 0.02939 | 21.87518399 |
|  | 4 | rs13119423 | 4 | 27850234 | -0.110370808 | 0.023980561 | 4.17427E-06 | 0.006111288 | 0.009649478 | 0.526542 | 21.17763672 |
|  | 5 | rs34865636 | 19 | 45793832 | -0.082856895 | 0.017673526 | 2.75628E-06 | 0.014257872 | 0.007105204 | 0.044821 | 21.97347019 |
|  | 6 | rs55682375 | 9 | 21808352 | -0.158559257 | 0.035199809 | 6.65141E-06 | 0.03555342 | 0.018042109 | 0.048787 | 20.28571586 |
|  | 7 | rs56001939 | 2 | 54363878 | -0.079585337 | 0.017042711 | 3.01555E-06 | 0.003486914 | 0.006705632 | 0.603087 | 21.80100297 |
|  | 8 | rs7255582 | 19 | 35718076 | -0.080016826 | 0.016918094 | 2.24897E-06 | -0.002682595 | 0.007379552 | 0.716189 | 22.36389741 |
| Species Oscillibacter_unclassified  (ebi-a-GSCT90027796) | 1 | rs10769221 | 11 | 5806426 | 0.140943307 | 0.027223119 | 2.25073E-07 | 0.003124115 | 0.012168263 | 0.797368 | 26.79790765 |
|  | 2 | rs11087270 | 20 | 18906606 | 0.088668244 | 0.01851595 | 1.67824E-06 | 0.001850287 | 0.007379044 | 0.802032 | 22.92619414 |
|  | 3 | rs12446334 | 16 | 81302009 | 0.093047565 | 0.020056903 | 3.49796E-06 | -0.017615241 | 0.008086062 | 0.02939 | 21.51642014 |
|  | 4 | rs13119423 | 4 | 27850234 | -0.110208568 | 0.023976273 | 4.29507E-06 | 0.006111288 | 0.009649478 | 0.526542 | 21.12297564 |
|  | 5 | rs34865636 | 19 | 45793832 | -0.082524169 | 0.017670366 | 3.00904E-06 | 0.014257872 | 0.007105204 | 0.044821 | 21.80514542 |
|  | 6 | rs55682375 | 9 | 21808352 | -0.158040017 | 0.035193514 | 7.10226E-06 | 0.03555342 | 0.018042109 | 0.048787 | 20.1602818 |
|  | 7 | rs56001939 | 2 | 54363878 | -0.07907059 | 0.017039664 | 3.47762E-06 | 0.003486914 | 0.006705632 | 0.603087 | 21.52760111 |
|  | 8 | rs7255582 | 19 | 35718076 | -0.080963762 | 0.016915069 | 1.69726E-06 | -0.002682595 | 0.007379552 | 0.716189 | 22.90453824 |
| Species Roseburia_hominis  (ebi-a-GSCT90027854) | 1 | rs215996 | 12 | 2719297 | -0.110749527 | 0.024036702 | 4.07505E-06 | 0.007261571 | 0.008434746 | 0.389281 | 21.22373183 |
|  | 2 | rs2225380 | 14 | 83605637 | -0.089414848 | 0.018862506 | 2.13351E-06 | 0.002573685 | 0.006860863 | 0.707579 | 22.46509312 |
|  | 3 | rs35004779 | 3 | 177893936 | 0.080729292 | 0.017551385 | 4.2331E-06 | -0.006966208 | 0.007406445 | 0.346929 | 21.15082042 |
|  | 4 | rs722241 | 16 | 13933400 | -0.103383953 | 0.022012063 | 2.64404E-06 | 0.002892812 | 0.008487382 | 0.73323 | 22.05324496 |
|  | 5 | rs7590468 | 2 | 68959471 | -0.089107502 | 0.018400335 | 1.2808E-06 | 0.009643353 | 0.006750023 | 0.153131 | 23.44578631 |
|  | 6 | rs7786467 | 7 | 1338707 | -0.096833817 | 0.021619233 | 7.49729E-06 | 0.012661504 | 0.010019983 | 0.206347 | 20.05678284 |
|  | 7 | rs854661 | 17 | 34354883 | 0.082735481 | 0.017155311 | 1.41605E-06 | -0.004242989 | 0.007650499 | 0.579181 | 23.25274085 |
| Species Rothia_mucilaginosa  (ebi-a-GSCT90027753) | 1 | rs1576176 | 1 | 98911950 | -0.168022077 | 0.038037807 | 9.99697E-06 | 0.006327936 | 0.007779402 | 0.415947 | 19.50695579 |
|  | 2 | rs17049177 | 2 | 129915467 | -0.266491849 | 0.05409938 | 8.39435E-07 | -0.001911826 | 0.01063343 | 0.857277 | 24.25889508 |
|  | 3 | rs616801 | 1 | 95011698 | 0.239716914 | 0.053312838 | 6.91071E-06 | -0.021469836 | 0.010128841 | 0.034051 | 20.2125678 |
|  | 4 | rs888195 | 12 | 2834412 | 0.161843405 | 0.034311016 | 2.39405E-06 | -0.010523175 | 0.006895762 | 0.127004 | 22.24388225 |
| Genus Pseudoflavonifractor  (ebi-a-GSCT90027706) | 1 | rs10236678 | 7 | 134549927 | 0.13208294 | 0.028060401 | 2.51273E-06 | -0.021162351 | 0.007422205 | 0.004369 | 22.15100501 |
|  | 2 | rs11074766 | 16 | 26304777 | 0.129592418 | 0.028421828 | 5.1249E-06 | -0.000900405 | 0.00847668 | 0.915347 | 20.78465917 |
|  | 3 | rs1182140 | 7 | 2964863 | -0.140384223 | 0.030959885 | 5.777E-06 | -0.013990411 | 0.009943502 | 0.159414 | 20.55538661 |
|  | 4 | rs4424244 | 8 | 74434869 | 0.121141343 | 0.025352016 | 1.76721E-06 | -0.003113843 | 0.006883069 | 0.651025 | 22.82693003 |
|  | 5 | rs6778836 | 3 | 168587709 | -0.125347415 | 0.026692431 | 2.65317E-06 | 0.00374199 | 0.007776365 | 0.63041 | 22.0466309 |
|  | 6 | rs75202637 | 4 | 163822623 | 0.261817734 | 0.057915131 | 6.16315E-06 | -0.008897465 | 0.019148759 | 0.642197 | 20.4315713 |
|  | 7 | rs7790846 | 7 | 50424061 | -0.120812267 | 0.026652853 | 5.82047E-06 | 0.009454168 | 0.007251302 | 0.192305 | 20.5410384 |
|  | 8 | rs7990301 | 13 | 34382060 | 0.110457283 | 0.02373951 | 3.27318E-06 | 0.003412172 | 0.007259846 | 0.638371 | 21.64377193 |
|  | 9 | rs9290954 | 3 | 190548613 | -0.121915635 | 0.02734728 | 8.27073E-06 | 0.012284239 | 0.007858628 | 0.11801 | 19.86910304 |
| Species Desulfovibrio_piger  (ebi-a-GSCT90027815) | 1 | rs10030640 | 4 | 18943713 | -0.128383971 | 0.028083241 | 4.84127E-06 | 0.003779847 | 0.007500172 | 0.614251 | 20.89367776 |
|  | 2 | rs196229 | 10 | 121459517 | -0.125690599 | 0.028342701 | 9.22123E-06 | -0.020563997 | 0.006796855 | 0.002491 | 19.66123781 |
|  | 3 | rs4854535 | 2 | 69180661 | 0.18056097 | 0.037784559 | 1.76435E-06 | 0.011168401 | 0.010296444 | 0.278059 | 22.83004576 |
|  | 4 | rs61818112 | 1 | 158039727 | 0.255213243 | 0.05774923 | 9.90047E-06 | 0.009883002 | 0.013721112 | 0.471387 | 19.52548112 |
|  | 5 | rs75364852 | 18 | 60781820 | 0.138788505 | 0.031376256 | 9.71772E-06 | 0.013449153 | 0.009183227 | 0.14304 | 19.56106454 |
|  | 6 | rs77902968 | 2 | 233883133 | -0.271803246 | 0.057994097 | 2.77591E-06 | 0.017468533 | 0.015704353 | 0.26597 | 21.95985285 |

**Supplementary Table 3.** MR results of causal links between the gut microbiome and AD risk (P <1 × 10^-5^).

| Classification | Nsnp | Methods | Beta | SE | OR (95% CI) | P-value | Horizontal pleiotropy | | Heterogeneity | |
| --- | --- | --- | --- | --- | --- | --- | --- | --- | --- | --- |
|  |  |  |  |  |  |  | MR-Egger Intercept test Intercept P-value | MR-PRESSO global test  P-value | | Cochran’s Q methods test  P-value |
| Order  Actinomycetales  (ebi-a-GSCT90027735) | 3 | MR Egger | -0.321 | 0.201 | 0.73 (0.49-1.07) | 0.356 | 0.039 0.437 | NA | | 0.458 |
|  |  | Weighted median | -0.065 | 0.037 | 0.94 (0.87-1.01) | 0.075 |  |  |  |  |
|  |  | Inverse variance weighted | -0.079 | 0.028 | 0.92 (0.88-0.98) | 0.004 |  |  |  |  |
|  |  | Weighted mode | -0.058 | 0.044 | 0.94 (0.87-1.03) | 0.32 |  |  |  |  |
|  |  | Simple mode | -0.055 | 0.042 | 0.95 (0.87-1.03) | 0.317 |  |  |  |  |
| Order  Pasteurellales  (ebi-a-GSCT90027746) | 3 | MR Egger | -0.051 | 0.211 | 0.95 (0.63-1.44) | 0.848 | 0.023 0.669 | NA | | 0.560 |
|  |  | Weighted median | 0.068 | 0.039 | 1.07 (0.99-1.16) | 0.081 |  |  |  |  |
|  |  | Inverse variance weighted | 0.068 | 0.031 | 1.07 (1.01-1.14) | 0.027 |  |  |  |  |
|  |  | Weighted mode | 0.076 | 0.048 | 1.08 (0.98-1.18) | 0.255 |  |  |  |  |
|  |  | Simple mode | 0.085 | 0.049 | 1.09 (0.99-1.20) | 0.227 |  |  |  |  |
| Family  Micrococcaceae  (ebi-a-GSCT90027661) | 6 | MR Egger | 0.008 | 0.117 | 1.01 (0.80-1.27) | 0.95 | -0.014 0.596 | 0.178 | | 0.113 |
|  |  | Weighted median | -0.043 | 0.026 | 0.96 (0.91-1.01) | 0.093 |  |  |  |  |
|  |  | Inverse variance weighted | -0.058 | 0.018 | 0.94 (0.91-0.98) | 0.001 |  |  |  |  |
|  |  | Weighted mode | -0.035 | 0.037 | 0.97 (0.90-1.04) | 0.389 |  |  |  |  |
|  |  | Simple mode | -0.038 | 0.038 | 0.96 (0.89-1.04) | 0.368 |  |  |  |  |
| Family  Pasteurellaceae  (ebi-a-GSCT90027685) | 3 | MR Egger | -0.051 | 0.211 | 0.95 (0.63-1.44) | 0.849 | 0.022 0.669 | NA | | 0.560 |
|  |  | Weighted median | 0.068 | 0.039 | 1.07 (0.99-1.16) | 0.081 |  |  |  |  |
|  |  | Inverse variance weighted | 0.068 | 0.031 | 1.07 (1.01-1.14) | 0.027 |  |  |  |  |
|  |  | Weighted mode | 0.076 | 0.048 | 1.08 (0.98-1.18) | 0.255 |  |  |  |  |
|  |  | Simple mode | 0.085 | 0.049 | 1.09 (0.99-1.20) | 0.227 |  |  |  |  |
| Family  Oscillospiraceae  (ebi-a-GSCT90027675) | 8 | MR Egger | -0.121 | 0.186 | 0.89 (0.62-1.27) | 0.538 | 0.005 0.782 | 0.308 | | 0.266 |
|  |  | Weighted median | -0.045 | 0.044 | 0.96 (0.88-1.04) | 0.31 |  |  |  |  |
|  |  | Inverse variance weighted | -0.069 | 0.031 | 0.93 (0.88-0.99) | 0.029 |  |  |  |  |
|  |  | Weighted mode | -0.004 | 0.065 | 1.00 (0.88-1.13) | 0.958 |  |  |  |  |
|  |  | Simple mode | -0.004 | 0.077 | 1.00 (0.86-1.16) | 0.964 |  |  |  |  |
| Family  Burkholderiales_noname  (ebi-a-GSCT90027680) | 13 | MR Egger | 0.019 | 0.079 | 1.02 (0.87-1.19) | 0.814 | 0.003 0.783 | 0.872 | | 0.850 |
|  |  | Weighted median | 0.048 | 0.021 | 1.05 (1.01-1.09) | 0.023 |  |  |  |  |
|  |  | Inverse variance weighted | 0.041 | 0.016 | 1.04 (1.01-1.07) | 0.011 |  |  |  |  |
|  |  | Weighted mode | 0.069 | 0.038 | 1.07 (0.99-1.15) | 0.094 |  |  |  |  |
|  |  | Simple mode | 0.068 | 0.037 | 1.07 (1.00-1.15) | 0.088 |  |  |  |  |
| Genus  Rothia  (ebi-a-GSCT90027687) | 6 | MR Egger | 0.008 | 0.118 | 1.01 (0.80-1.27) | 0.95 | -0.014 0.597 | 0.179 | | 0.113 |
|  |  | Weighted median | -0.044 | 0.026 | 0.96 (0.91-1.01) | 0.092 |  |  |  |  |
|  |  | Inverse variance weighted | -0.058 | 0.018 | 0.94 (0.91-0.98) | 0.001 |  |  |  |  |
|  |  | Weighted mode | -0.035 | 0.037 | 0.97 (0.90-1.04) | 0.389 |  |  |  |  |
|  |  | Simple mode | -0.038 | 0.038 | 0.96 (0.89-1.04) | 0.368 |  |  |  |  |
| Genus  Burkholderiales_noname  (ebi-a-GSCT90027725) | 13 | MR Egger | 0.019 | 0.079 | 1.02 (0.87-1.19) | 0.818 | 0.004 0.779 | 0.872 | | 0.850 |
|  |  | Weighted median | 0.048 | 0.021 | 1.05 (1.01-1.09) | 0.023 |  |  |  |  |
|  |  | Inverse variance weighted | 0.041 | 0.016 | 1.04 (1.01-1.07) | 0.011 |  |  |  |  |
|  |  | Weighted mode | 0.069 | 0.038 | 1.07 (0.99-1.16) | 0.097 |  |  |  |  |
|  |  | Simple mode | 0.068 | 0.037 | 1.07 (1.00-1.15) | 0.087 |  |  |  |  |
| Genus  Oscillibacter  (ebi-a-GSCT90027714) | 9 | MR Egger | -0.136 | 0.167 | 0.87 (0.63-1.21) | 0.443 | 0.006 0.712 | 0.381 | | 0.337 |
|  |  | Weighted median | -0.049 | 0.041 | 0.95 (0.88-1.03) | 0.234 |  |  |  |  |
|  |  | Inverse variance weighted | -0.073 | 0.03 | 0.93 (0.88-0.99) | 0.014 |  |  |  |  |
|  |  | Weighted mode | -0.007 | 0.066 | 0.99 (0.87-1.13) | 0.923 |  |  |  |  |
|  |  | Simple mode | -0.009 | 0.072 | 0.99 (0.86-1.14) | 0.903 |  |  |  |  |
| Genus  Pseudoflavonifractor  (ebi-a-GSCT90027706) | 9 | MR Egger | -0.034 | 0.165 | 0.97 (0.70-1.34) | 0.843 | -0.001 0.964 | 0.221 | | 0.187 |
|  |  | Weighted median | -0.029 | 0.03 | 0.97 (0.91-1.03) | 0.335 |  |  |  |  |
|  |  | Inverse variance weighted | -0.041 | 0.021 | 0.96 (0.92-1.00) | 0.049 |  |  |  |  |
|  |  | Weighted mode | -0.03 | 0.045 | 0.97 (0.89-1.06) | 0.526 |  |  |  |  |
|  |  | Simple mode | -0.03 | 0.045 | 0.97 (0.89-1.06) | 0.527 |  |  |  |  |
| Genus  Collinsella  (ebi-a-GSCT90027690) | 8 | MR Egger | -0.055 | 0.173 | 0.95 (0.67-1.33) | 0.761 | -0.002 0.914 | 0.726 | | 0.666 |
|  |  | Weighted median | -0.067 | 0.038 | 0.93 (0.87-1.01) | 0.078 |  |  |  |  |
|  |  | Inverse variance weighted | -0.074 | 0.028 | 0.93 (0.88-0.98) | 0.009 |  |  |  |  |
|  |  | Weighted mode | -0.07 | 0.049 | 0.93 (0.85-1.03) | 0.201 |  |  |  |  |
|  |  | Simple mode | -0.079 | 0.058 | 0.92 (0.82-1.04) | 0.217 |  |  |  |  |
| Species  Burkholderiales_bacterium_1_1_47  (ebi-a-GSCT90027809) | 13 | MR Egger | 0.018 | 0.078 | 1.02 (0.87-1.19) | 0.819 | 0.004 0.778 | 0.872 | | 0.850 |
|  |  | Weighted median | 0.048 | 0.021 | 1.05 (1.01-1.09) | 0.023 |  |  |  |  |
|  |  | Inverse variance weighted | 0.041 | 0.016 | 1.04 (1.01-1.07) | 0.011 |  |  |  |  |
|  |  | Weighted mode | 0.069 | 0.038 | 1.07 (0.99-1.15) | 0.094 |  |  |  |  |
|  |  | Simple mode | 0.068 | 0.037 | 1.07 (1.00-1.15) | 0.088 |  |  |  |  |
| Species  Parabacteroides_merdae  (ebi-a-GSCT90027769) | 8 | MR Egger | -0.155 | 0.124 | 0.86 (0.67-1.09) | 0.257 | 0.011 0.476 | 0.336 | | 0.246 |
|  |  | Weighted median | -0.044 | 0.04 | 0.96 (0.88-1.04) | 0.273 |  |  |  |  |
|  |  | Inverse variance weighted | -0.065 | 0.029 | 0.94 (0.88-0.99) | 0.028 |  |  |  |  |
|  |  | Weighted mode | -0.03 | 0.048 | 0.97 (0.88-1.07) | 0.547 |  |  |  |  |
|  |  | Simple mode | -0.063 | 0.057 | 0.94 (0.84-1.05) | 0.311 |  |  |  |  |
| Species  Oscillibacter_unclassified  (ebi-a-GSCT90027796) | 8 | MR Egger | -0.112 | 0.189 | 0.89 (0.62-1.29) | 0.575 | 0.004 0.821 | 0.307 | | 0.261 |
|  |  | Weighted median | -0.045 | 0.045 | 0.96 (0.88-1.04) | 0.315 |  |  |  |  |
|  |  | Inverse variance weighted | -0.068 | 0.031 | 0.93 (0.88-0.99) | 0.03 |  |  |  |  |
|  |  | Weighted mode | -0.003 | 0.066 | 1.00 (0.88-1.13) | 0.969 |  |  |  |  |
|  |  | Simple mode | -0.004 | 0.077 | 1.00 (0.86-1.16) | 0.961 |  |  |  |  |
| Species  Roseburia_hominis  (ebi-a-GSCT90027854) | 7 | MR Egger | -0.018 | 0.3 | 0.98 (0.54-1.77) | 0.954 | -0.005 0.874 | 0.980 | | 0.976 |
|  |  | Weighted median | -0.062 | 0.041 | 0.94 (0.87-1.02) | 0.126 |  |  |  |  |
|  |  | Inverse variance weighted | 0.032 | 0.032 | 0.93 (0.88-0.99) | 0.032 |  |  |  |  |
|  |  | Weighted mode | -0.047 | 0.058 | 0.95 (0.85-1.07) | 0.447 |  |  |  |  |
|  |  | Simple mode | -0.05 | 0.054 | 0.95 (0.86-1.06) | 0.392 |  |  |  |  |
| Species  Rothia_mucilaginosa  (ebi-a-GSCT90027753) | 4 | MR Egger | 0.013 | 0.117 | 1.01 (0.81-1.27) | 0.92 | -0.012 0.661 | 0.455 | | 0.381 |
|  |  | Weighted median | -0.047 | 0.027 | 0.95 (0.90-1.01) | 0.086 |  |  |  |  |
|  |  | Inverse variance weighted | -0.045 | 0.021 | 0.96 (0.92-1.00) | 0.035 |  |  |  |  |
|  |  | Weighted mode | -0.063 | 0.04 | 0.94 (0.87-1.02) | 0.214 |  |  |  |  |
|  |  | Simple mode | -0.063 | 0.042 | 0.94 (0.86-1.02) | 0.226 |  |  |  |  |
| Species  Desulfovibrio_piger  (ebi-a-GSCT90027815) | 6 | MR Egger | -0.092 | 0.113 | 0.91 (0.73-1.14) | 0.463 | 0.023 0.275 | 0.086 | | 0.0571 |
|  |  | Weighted median | 0.045 | 0.034 | 1.05 (0.98-1.12) | 0.185 |  |  |  |  |
|  |  | Inverse variance weighted | 0.045 | 0.023 | 1.05 (1.00-1.10) | 0.053 |  |  |  |  |
|  |  | Weighted mode | 0.045 | 0.051 | 1.05 (0.95-1.16) | 0.422 |  |  |  |  |
|  |  | Simple mode | 0.058 | 0.057 | 1.06 (0.95-1.19) | 0.362 |  |  |  |  |
